# Metallochaperone UreG serves as a new target for design of urease inhibitor: A novel strategy for development of antimicrobials

**DOI:** 10.1371/journal.pbio.2003887

**Published:** 2018-01-10

**Authors:** Xinming Yang, Mohamad Koohi-Moghadam, Runming Wang, Yuen-Yan Chang, Patrick C. Y. Woo, Junwen Wang, Hongyan Li, Hongzhe Sun

**Affiliations:** 1 Department of Chemistry, The University of Hong Kong, Hong Kong; 2 Center for Genomic Sciences, The University of Hong Kong, Hong Kong; 3 Center for Individualized Medicine & Department of Health Sciences Research, Mayo Clinic, Scottsdale, Arizona, United States of America; 4 Department of Microbiology, The University of Hong Kong, Hong Kong; 5 State Key Laboratory of Emerging Infectious Diseases, The University of Hong Kong, Hong Kong; 6 The Research Centre of Infection and Immunology, Li Ka Shing Faculty of Medicine, The University of Hong Kong, Hong Kong; 7 Department of Biomedical Informatics, Arizona State University, Scottsdale, Arizona, United States of America; Brigham and Women's Hospital, United States of America

## Abstract

Urease as a potential target of antimicrobial drugs has received considerable attention given its versatile roles in microbial infection. Development of effective urease inhibitors, however, is a significant challenge due to the deeply buried active site and highly specific substrate of a bacterial urease. Conventionally, urease inhibitors are designed by either targeting the active site or mimicking substrate of urease, which is not efficient. Up to now, only one effective inhibitor—acetohydroxamic acid (AHA)—is clinically available, but it has adverse side effects. Herein, we demonstrate that a clinically used drug, colloidal bismuth subcitrate, utilizes an unusual way to inhibit urease activity, i.e., disruption of urease maturation process via functional perturbation of a metallochaperone, UreG. Similar phenomena were also observed in various pathogenic bacteria, suggesting that UreG may serve as a general target for design of new types of urease inhibitors. Using *Helicobacter pylori* UreG as a showcase, by virtual screening combined with experimental validation, we show that two compounds targeting UreG also efficiently inhibited urease activity with inhibitory concentration (IC)_50_ values of micromolar level, resulting in attenuated virulence of the pathogen. We further demonstrate the efficacy of the compounds in a mammalian cell infection model. This study opens up a new opportunity for the design of more effective urease inhibitors and clearly indicates that metallochaperones involved in the maturation of important microbial metalloenzymes serve as new targets for devising a new type of antimicrobial drugs.

## Author summary

Urease, a metalloenzyme that catalyzes the hydrolysis of urea, plays important roles in the survival and virulence of many microbial pathogens, and has long been considered an important drug target for the development of novel antimicrobials. However, its deeply buried active site and highly specific substrate of bacterial urease make it very challenging to design effective urease inhibitors by conventional approaches. In this study, we reveal that a bismuth-based drug (colloidal bismuth subcitrate) inhibits urease activity in an unusual way. This drug binds the urease accessary protein UreG and inhibits its GTPase activity, thus perturbing nickel insertion into the apo-urease, a process called urease maturation. UreG is therefore proposed as an alternative target for the development of urease inhibitors. Using *H*. *pylori* UreG as an example, combined with virtual screening and experimental validation, we further show that several small molecules that bind and functionally disrupt UreG could indeed inhibit urease activity in bacteria and in a cell infection model and possess potent antimicrobial activity. In summary, we discovered metallochaperone UreG as a new target for the design of urease inhibitors. Such a strategy should have a broad application in the development of metalloenzyme inhibitors.

## Introduction

Enzymes have been proven to serve as important drug targets, and enzyme inhibitors are among the most successful drugs [1]. One dominant strategy for enzyme-targeted drug design lies in the discovery or synthesis of structure analogues of substrates that resemble the enzyme’s reactivity [2]. However, such a strategy might be restrained if the active sites of enzymes are not solvent exposed or if the substrates of enzymes are highly specific. This is the case for urease, an enzyme that catalyzes the hydrolysis of urea in plants, fungi, and many pathogenic bacteria into ammonia and carbonic acid [3]. X-ray structures of ureases reveal that the conserved active sites consist of a bis-μ-hydroxo dimeric nickel center deeply buried in the supramolecular assembly [4,5]. Moreover, urease has a highly specific substrate, i.e., urea [5]. This makes it very challenging for the development of urease inhibitors by conventional methods. Alternative strategies are urgently needed to design urease inhibitors.

Urease has been recognized as a general microbial virulence factor [3]. *H*. *pylori* relies on urease to invade the host epithelial cells and ensure successful colonization [6]. The increased pH of the urinary system led by urease from *Proteus mirabilis* may result in the formation of urinary and kidney stones, catheter occlusion, and kidney infection [7]. Moreover, survival of *Mycobacterium* species in the phagolysosomes of infected cells depends on its urease activity to neutralize the local acid to avoid the destructive effect caused by cathepsins [8]. Recently, the roles of urease during fungal infections in humans are beginning to be recognized. For example, urease from *Cryptococcus neoformans* has been observed to facilitate the crossing of the blood–brain barrier in a mice model [9].

Given the importance of urease in the virulence of microbial pathogens, urease is thus considered one of the most important targets in the development of drugs, especially for the treatment of gastric and urinary infections [10,11]. Up to now, urease inhibitors were designed exclusively by either attacking the metallocenter or mimicking the substrate of ureases [10,12,13]. In spite of enormous efforts being made, only one compound—acetohydroxamic acid (AHA)—with anti-urease activity has been used clinically for the treatment of urinary tract infections together with antibiotics [13,14]. Unfortunately, adverse side effects, including inhibition of the biosynthesis of bone marrow, depressed DNA synthesis, and malformation of embryos at high doses, have been reported [15]. The potential of urease as a target for antimicrobial agents, therefore, has not yet been fully explored.

As apo-urease synthesized in microbes is inactive, urease exerts its function only if nickel is inserted into its active site [16]. The assembly of the active site of bacterial ureases, a process also called urease maturation, is a complex and guanosine-5'-triphosphate (GTP)-dependent process, involving the cooperative actions of several accessory proteins [16]. For example, the canonical urease system in *H*. *pylori* requires UreE, UreF, UreG, and UreH to facilitate activation of the UreAB apo-enzyme. It has been proposed that nickel translocation from UreE to UreG is triggered by the formation of UreE-UreG (2E-2G) complex. Subsequently, Ni-UreG binds to UreF/H to form a supercomplex as an apo-urease/UreF-H-G, in which the final step of nickel insertion into the apo-urease is completed upon the GTP hydrolysis by UreG [17]. Homologs of UreF, UreG, and UreH/D can also be found in urease-producing eukaryotes [16], indicating the maturation of urease is relatively conserved among different species. We therefore envision that disruption of urease maturation process to inactivate its function might serve as a superior strategy for the design of new types of inhibitors.

Urease is known to be a major contributor to the pathogenesis of *H*. *pylori*, a transmissible human pathogen strongly related to gastrointestinal diseases including gastritis, peptic ulcers, and even stomach cancer [18]. The bismuth-based quadruple therapy has been used clinically as first-line therapy and surprisingly shown excellent success in eradication of *H*. *pylori*, even for antibiotic resistance strains [18–21]. However, the mechanism of action of bismuth drugs is not fully understood [22]. Although it has been hypothesized that bismuth drugs may exert antimicrobial action via inhibition of urease [23], our previous study on jack bean and *Klebsiella aerogenes* ureases showed that bismuth compounds exert very low in vitro activity towards inhibition of urease and could not displace nickel from urease [24]. Given the critical role of urease in the survival and pathogenesis of the pathogen [18], it is highly possible that bismuth inhibits urease activity via an alternative approach rather than direct perturbation on the active site. The knowledge of how bismuth exerts its inhibition on *H*. *pylori* urease may promote discovery of new targets for the development of antimicrobial agents.

In this report, we first elucidated the mechanism of action of a clinically used bismuth drug, colloidal bismuth subcitrate (CBS), on inhibition of urease of *H*. *pylori* with the aim of searching for new targets for design of urease inhibitors. We demonstrate that the bismuth drug inhibited urease activity in *H*. *pylori* through binding to the urease accessary protein UreG, thus perturbing its GTPase activity and leading to subsequent disruption of urease maturation. Such a phenomenon was also found in other pathogenic bacteria. Using *H*. *pylori* UreG as a target, two compounds were identified by virtual screening and subsequently verified to attenuate urease activity attributable to the disturbance of essential chaperones and exhibited comparable or even better antimicrobial activity towards *H*. *pylori* compared to bismuth drug CBS or AHA with IC_50(urease)_ values at micromolar levels. The efficacy of these compounds was further confirmed in a mammalian cell infection model. Overall, our studies offer a new strategy for design of urease inhibitors, as well as a novel paradigm for rational design of a new type of antimicrobial.

## Results

### Bismuth inhibits urease activity through binding and functional perturbation of UreG

**Fig 1:**
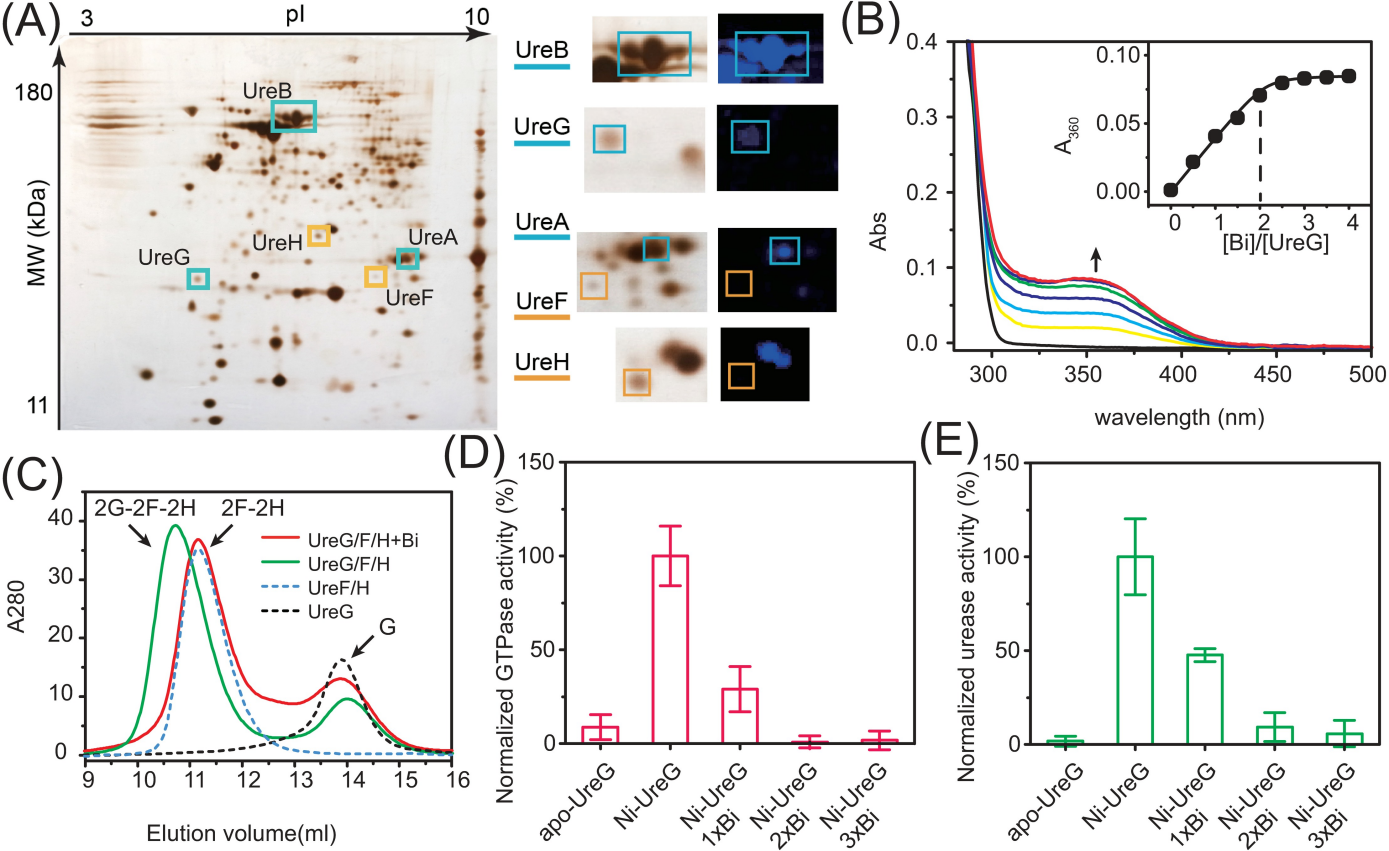
Binding of bismuth (III) to UreG disrupts urease maturation. (A) Analysis of Bi-binding proteins in live *H*. *pylori* cells by 2-DE. The proteins that bind to Bi(III) are highlighted in blue squares on the SDS-PAGE (as UreA, UreB and UreG), while those that do not bind Bi(III) are in orange squares (as UreF, UreH). (B) UV-vis spectra of UreG in HEPES buffer titrated with BiNTA with the titration curve shown in the inset. (C) Gel filtration profiles of protein complexes with or without Bi(III). In the presence of Mg(II) and GTP (without Ni(II)), 2G-2F-2H complex was eluted at approximately 10.5 ml. Incubation with two molar equivalents of Bi(III) resulted in the dissociation of UreG from 2F-2H complex. An excess amount of UreG was used and eluted as a monomer at approximately 14 ml (green curve). (D) Activity of UreG as a GTPase. The activity of apo- or Ni-bound UreG was examined, whereas the Ni-UreG was incubated with varying concentrations of Bi(III) ions to study the effects of Bi(III) ions on UreG activity. (E) Analysis of the effects of metal–UreG interaction on the urease activity. Apo- or metal-treated UreG was supplemented to the cell lysate of *Escherichia coli*-expressing urease gene cluster, excluding *ureG* gene, prior to analysis (incubation for 40 min). The activities of UreG or urease in the samples without CBS treatment were set as 100%; the activities of the negative control (without addition of UreG into the reactions) were set as 0. The underlying data for Fig 1D and 1E can be found in S1 Data. 2-DE, 2-dimensional gel electrophoresis; CBS, colloidal bismuth subcitrate.

Given the critical role of urease in the pathogenesis of *H*. *pylori* and the success of bismuth-based drugs in the treatment of *H*. *pylori* infection, urease has been proposed to be a key target of bismuth drugs [23]. However, previous studies showed that bismuth exhibited trivial effects on urease activity even at high concentration (up to several mM level) [24–26], which is unlikely to be accountable for its in vivo activity. We hypothesized that Bi(III) might inhibit urease activity indirectly, such as through disruption of urease maturation. To search which urease accessory proteins bound to Bi(III), we employed a homemade fluorescent probe Bi-*NTA-AC* [27], similar to Ni-*NTA-AC* [28], that can rapidly enter live cells to fluorescently label metal binding proteins in our recent report; the labelled proteins can be identified subsequently by proteomics upon photoactivation of arylazide of the probe [29]. Urease-related proteins lightened up by the probe are shown in Fig 1A. UreA and UreB, the two subunits of urease, were fluorescently labeled by Bi-*NTA-AC*, indicating both UreA and UreB bound to Bi(III) in *H*. *pylori* cells, consistent with our previous reports [25,26]. Among the urease accessory proteins, UreG exhibited blue fluorescence on the gel, indicating that UreG interacted with Bi(III) in *H*. *pylori* cells, while UreF and UreH were not fluorescently labeled and UreE was not identified both in silver-stained gel and fluorescence image, possibly due to low expression level in the bacterium.

We further monitored the interactions of Bi(III) with these chaperones in vitro by ultraviolet-visible (UV-vis) spectroscopy. Titration of up to four molar equivalents of Bi(III) (as Bi nitrilotriacetic acid [NTA]) into solutions of apo-UreE or -UreFH complex led to negligible changes in the UV spectra (S1 Fig), in agreement with in vivo data that UreE and UreF/H do not interact with Bi(III). In contrast, upon addition of Bi(III) to apo-UreG solution, a peak centered at about 350 nm, assignable to the π(S)(Cys)→Bi(III) ligand-to-metal charge transfer (LMCT) [30], appeared, increased its intensities, and levelled off at a molar ratio of [BiNTA]/[UreG] of ca. 2 (Fig 1B), indicating that two Bi(III) ions bound to each UreG monomer. The titration data were nonlinearly fitted via Ryan–Weber equation, and by taking account of Bi-NTA binding affinity [30], an apparent dissociation constant (*K*_d_) of Bi(III) to UreG was determined to be 3.1 × 10^−24^ M. As UreG contains only two cysteine residues on the protein surface, Cys48 and Cys66 [31], we prepared either single (UreG-C48A or UreG-C66A) or double mutants (UreG-C48C66A) of UreG and performed similar experiments. As expected, titration of Bi(III) into solutions of either UreG-C48A or UreG-C66A resulted in increases in intensities of the peak centered at approximately 350 nm with plateaus reached at [BiNTA]/[Protein] of 1:1. In contrast, titration of Bi(III) into UreG-C48C66A led to no absorbance at 350 nm (S1 Fig), indicating that both Cys48 and Cys66 of UreG participated in the binding of Bi(III).

As Cys66 is involved in the binding of Ni(II) [31], it is highly possible that Bi(III) perturbs the Ni(II) coordination to the protein. Indeed, titration of Ni(II) (as NiSO_4_) into the Bi-UreG solution resulted in no changes in the UV spectra in the presence of GTP and Mg(II) (S2 Fig), suggesting that the metal binding site became inaccessible to Ni(II) once it was occupied by Bi(III). Unexpectedly, stepwise addition of up to three molar equivalents of Bi(III) to Ni-UreG did not suppress the characteristic peak at approximately 337 nm (π(S)(Cys)→Ni(II) LMCT), while the peak at approximately 350 nm (π(S)(Cys)→Bi(III) LMCT) remained undetectable, indicating the lack of Bi(III) coordination to UreG protein when the metal binding site was preloaded with Ni(II) (S2 Fig). However, in the presence of GTPase-activating element KHCO_3_ [17], gradual addition of Bi(III) to UreG solution led to a decrease in the intensity and complete disappearance of the peak at approximately 337 nm accompanied by the emergence of a peak at approximately 350 nm (S2 Fig), indicative of the simultaneous replacement of Ni(II) ions by Bi(III) from UreG protein. In contrast, titration of Bi(III) into Ni-UreG solution in the presence of KHCO_3_ and GTPγs (a nonhydrolyzable analogue of GTP) did not disturbed characteristic Ni-binding peak, while the typical Bi coordination peak at 350 nm was unnoticeable (S2 Fig). Taken together, we demonstrate that Bi(III) only disturbed UreG dimer at its GTPase transition state (i.e., in the presence of GTPase-activating elements), but not at its stable Ni, GTP-bound state.

Binding of Bi(III) to UreG also induced the tertiary structural changes of Ni-bound UreG in the presence of KHCO_3_ as revealed by gel filtration chromatography. Incubation of Bi(III) with Ni-UreG (in the presence of KHCO_3_) led to a decrease in the intensity of UreG dimer peak, which was eluted at approximately 13 ml, and appearance of new peaks at elution volumes of approximately 18 ml and approximately 6 ml, with the former assignable to GTP molecule and the latter to high molecular weight oligomer of the protein (S3 Fig).

We next examined the effects of Bi-UreG interaction on the formation of UreE-UreG and UreG-UreFH complexes, which are crucial for the Ni(II) delivery into the Ni-containing active site of urease [17,31]. A previous study revealed that the active complex UreE-UreG was assembled by an UreE dimer and an UreG dimer (i.e., 2E-2G), while UreG-FH complex was assembled as a dimer of UreG/F/H (i.e., 2G-2F-2H) [17,31]. Incubation of Bi(III) with UreE-UreG led to the complete disappearance of 2E-2G peak, which was eluted at approximately 11.8 ml in gel filtration chromatography, accompanied by the appearance of a new broad peak at elution volumes of approximately 13 ml (S3 Fig). Notably, supplementation of Bi(III) had no effects on the elution volumes of UreE dimer (S4 Fig), implying that Bi(III) disrupted the formation of 2E-2G complex through targeting UreG. Similarly, UreG was dissociated from 2G-2F-2H complex without disrupting the 2F-2H complex upon Bi(III) incubation (Fig 1C). These data confirm that binding of Bi(III) to UreG disturbed the protein–protein complexation, which is essential for urease maturation.

We then investigated the effects of Bi-UreG interaction on the enzymatic activity of UreG by carrying out GTPase assay in the absence and presence of Bi(III). As shown in Fig 1D, a very low GTPase activity was observed for apo-UreG; upon incubation of apo-UreG with Ni(II), maximum GTPase activity was achieved, confirming the necessity of Ni(II) ions for the function of UreG as a GTPase. Addition of equimolar amounts of Bi(III) to Ni-UreG led to a drop in GTPase activity by 60%, and further addition of Bi(III) resulted in complete inhibition of the enzyme activity, signifying that binding of Bi(III) completely abolished GTPase activity of UreG (Fig 1D).

We subsequently evaluated the effects of binding of Bi(III) to UreG on the activity of urease using cell lysate of *E*. *coli* harboring plasmid pHP8080ΔG, which consists of the urease gene cluster except *ureG* (as *ureABIEFH*Δ*G*). Purified UreG protein with or without treatment of Ni(II) or Bi(III) ions was supplemented to the system sequentially. As shown in Fig 1E, the maximum urease activity (normalized to 100%) was achieved only when Ni-UreG was supplemented into the cell lysate. In contrast, no observable urease activity was found when apo-UreG was added. When stoichiometric amounts of Bi(III) ions were added to Ni-UreG, the urease activity was decreased by 50% and further addition of two or three molar equivalents of Bi(III) ions to Ni-UreG led to complete abolishment of urease activity (Fig 1E), demonstrating that the inactivation of urease is attributable to Bi(III) binding to UreG. We also investigated the influence of CBS on the GTPase and urease activity in bacterial cells. The *ureG* gene was complemented to *E*. *coli* cells harboring plasmid pHP8080ΔG, after which the GTPase and ureolytic activities of *E*. *coli* cells were monitored simultaneously. As shown in S5 Fig, treatment of *E*. *coli* with increasing amounts of CBS during bacterial growth resulted in the dramatic concerted declines in both the ureolytic and GTPase activities to approximately 20% compared with those without CBS treatment, implying that binding of Bi(III) to UreG abolished its enzymatic activity, leading to inactivation of urease through disruption of urease maturation in engineered bacterium.

### Bismuth disrupts urease maturation in pathogenic bacteria

**Fig 2:**
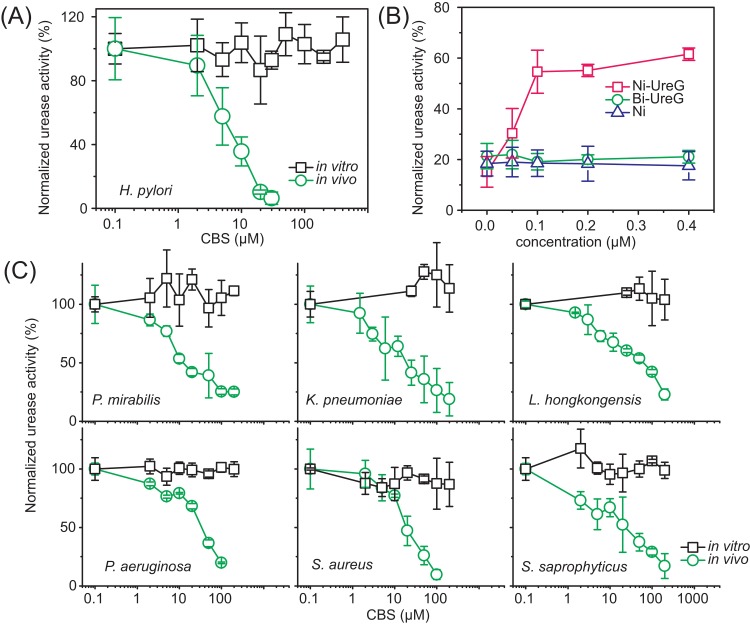
Effect of CBS on urease activity in pathogenic bacteria. (A) Urease activity of *H*. *pylori* with or without the supplementation of CBS to the bacterial culture (marked as in vivo in the figure, circles) and to the extracted enzymes (marked as in vitro in the figure, squares). (B) Recovery of urease activity upon supplementation of additional Ni-UreG, CBS-treated (two molar equivalents) Ni-UreG (Bi-UreG), and Ni ions alone into CBS-treated *H*. *pylori* cell lysate (incubation for 40 min). The starting sample with 20% urease activity was used. (C) Urease activity of *P*. *mirabilis*, *K*. *pneumoniae*, *Laribacter hongkongensis*, *Pseudomonas aeruginosa*, *Staphylococcus aureus*, and *S*. *saprophyticus*, with or without the supplementation of CBS to the bacterial culture (marked as in vivo in the figure, circles) and to the extracted enzymes (marked as in vitro in the figure, squares). Because zero is not defined on a log scale, the blank values have been entered as 0.1. For convenient comparison, the activities of urease in the samples without CBS treatment were set as 100%; the activities of the negative control (without addition of cell lysate into the reactions) were set as 0. The underlying data can be found in S1 Data. CBS, colloidal bismuth subcitrate.

We have shown that a Bi(III) drug (CBS) can inhibit urease activation at relatively low concentration (μM level) through functional disruption of chaperone UreG in vitro and in an engineered bacterium. We further investigated whether this is also the case in *H*. *pylori*. We supplemented various concentrations (below minimum inhibitory concentration [MIC]) [25] of CBS to the culture medium during *H*. *pylori* growth, then measured urease activity in live bacterial cells. Notably, a dramatic decline in the ureolytic activity of *H*. *pylori* urease in live bacterial cells was coupled with the increasing concentration of CBS, and a drop in urease activity by ca. 90% was observed at CBS concentration as low as 20 μM (Fig 2A). In contrast, incubation of CBS (up to 400 μM) with extracted *H*. *pylori* urease resulted in little inhibitory effect (Fig 2A).

We then measured the nickel content in *H*. *pylori* cells with or without supplementation of Bi(III) to culture medium. As shown in S6 Fig, only a slight decline in nickel content was noticed upon supplementation of CBS, indicating that Bi(III) did not influence the influx of Ni(II) into bacterial cells. We have shown previously that the expression levels of the urease system were not significantly altered upon treatment with Bi(III)-based drugs [25]. We reason that the urease extracted from bacterial lysate might have already been maturated, i.e., urease exists in the Ni-bound form, and in this case, Bi(III) cannot replace Ni(II) from the active site. On the other hand, a newly synthesized urease exists in an apo-form and is inactive, which requires urease accessary proteins for maturation. Binding of bismuth drugs to UreG may thus disrupt urease maturation in live bacterial cells. Indeed, when gradient amounts of Ni-bound urease accessory protein UreG were incubated with CBS-treated *H*. *pylori* cell lysate, the urease activity was recovered by about 60% (Fig 2B), corroborating the theory that the reduced ureolytic activity of urease is likely due to the Ni(II)-deficiency in the active site of enzyme. Notably, supplementation of Ni(II) ions alone or CBS-treated Ni-UreG to the bacterial cell lysate failed to restore the urease activity completely (Fig 2B), pinpointing that bismuth drugs inhibited urease activity via targeting accessory protein UreG in vivo.

Strikingly, similar phenomena were also observed when CBS was supplied to the culture of various bacterial species (either gram-negative or gram-positive, even some clinically isolated strains), including *P*. *mirabilis*, *K*. *pneumoniae*, *L*. *hongkongensis*, *P*. *aeruginosa*, *S*. *aureus*, and *S*. *saprophyticus* (Fig 2C). Importantly, CBS showed more potent efficiency than AHA towards inhibition of urease activity in all tested bacterial species (S7 Fig). Maturation of ureases from these bacteria also require similar accessary proteins, especially the Ni-dependent GTPase UreG is much conserved among all these bacterial species (S8 Fig). Inhibition of urease activity in vivo by bismuth drugs via disruption of urease maturation appears to be a general feature irrespective of the bacterial species. UreG might, therefore, serve as an alternative target for the design of potent urease inhibitors.

### UreG serves as a novel target for design of new antimicrobial agents

**Fig 3:**
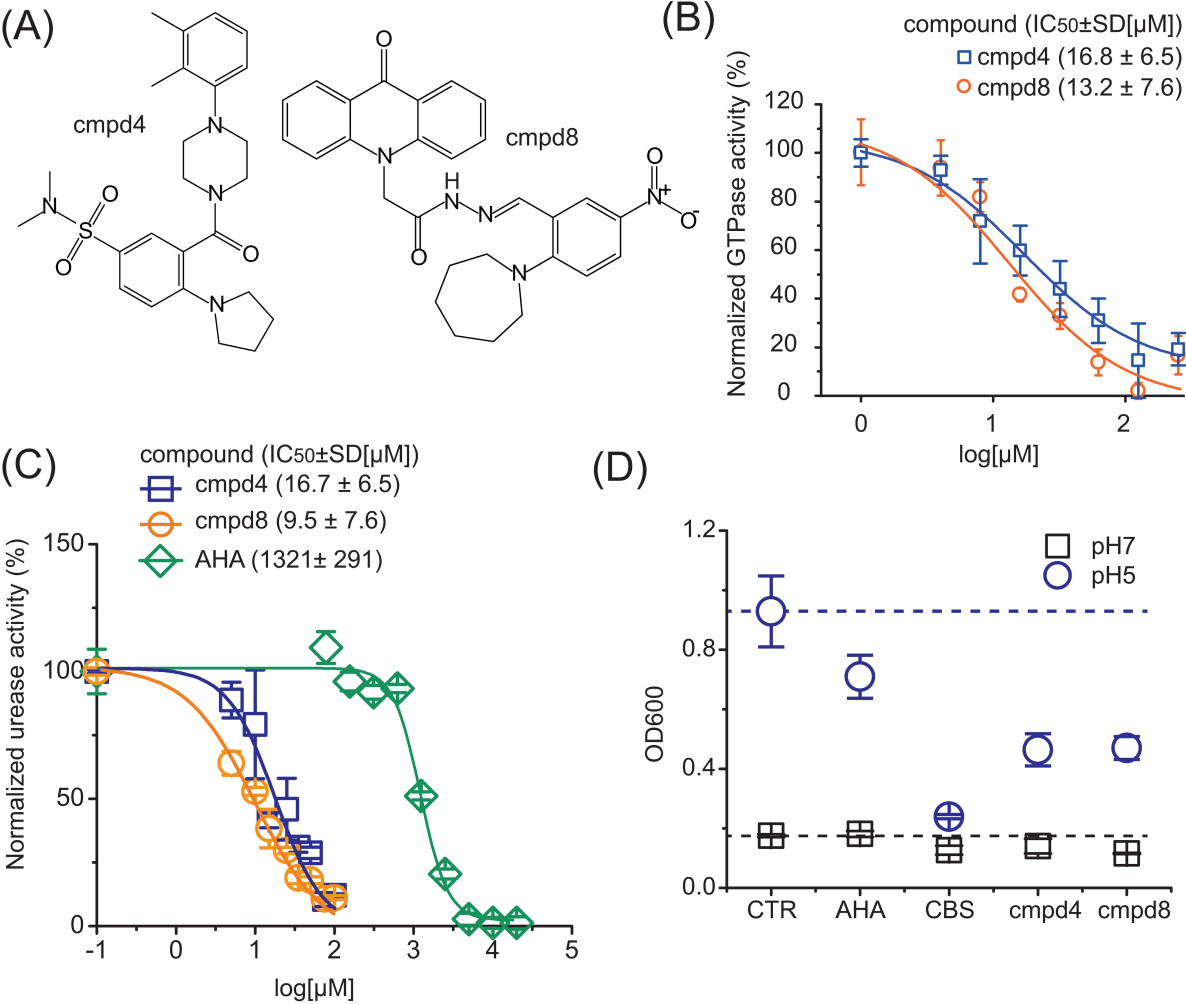
Development of small compounds as urease inhibitors via targeting UreG. (A) The structures of the representative hits, **cmpd4** and **cmpd8**. (B) Inhibition of GTPase activity of purified Ni-UreG by **cmpd4** and **cmpd8**. (C) Effect of **cmpd4** and **cmpd8** on urease activity of *H*. *pylori*. AHA is shown for comparison. As AHA attacks urease directly, AHA was incubated with extracted urease to investigate effect of AHA on urease activity. Because zero is not defined on a log scale, the blank values have been entered as 0.1. The activities of UreG or urease in the samples without **cmpd4/cmpd8** treatment were set as 100%; the activities of the negative control (without addition of UreG/extracted urease into the reactions) were set as 0. (D) The effect of AHA and UreG inhibitors (CBS, **cmpd4**, and **cmpd8**) on the growth of *H*. *pylori* in neutral and acidic medium. In the absence of inhibitors (control group), *H*. *pylori* exhibited excellent growth in the acidic brucella broth (OD_600_ = 0.93) compared with that in neutral medium (OD_600_ = 0.18). AHA, CBS, **cmpd4**, and **cmpd8** hindered the growth of *H*. *pylori* in acidic medium. The underlying data can be found in S1 Data. AHA, acetohydroxamic acid; CBS, colloidal bismuth subcitrate; CTR, control group.

As bismuth inhibits urease via disruption of its maturation through perturbation of UreG, we envision that small molecules that functionally disrupt the GTPase activity of UreG could potentially be effective inhibitors of urease in microbes. To validate this hypothesis, we performed a virtual screening using AutoDock Vina [32]. As bismuth inhibits UreG activity by targeting the nickel binding site, which is located on the surface of UreG, we first attempted to find small molecules that could bind to this metal binding site; unfortunately, our initial screening resulted in no hits. We then turned our attention to search for small molecules that may block the guanine nucleotide binding pocket, which is located next to the nickel binding site, given that the contact between nucleotide binding pocket and metal binding site is essential for the nickel insertion into apo-urease [17,31]. UreG structure from *H*. *pylori* (PDB 4HI0) was used as the docking receptor. A set of 1,700 compounds collected from the databases PubChem [33] and BindingDB [34] were used for the screening. Three hundred of these compounds are active compounds as GTPase inhibitors based on the PubChem database [33], and 1,400 of them showed a potential interaction with a G-protein in different species based on BindingDB. We ranked the 1,700 compounds based on their docking scores. Out of this screening and based on structural features, physical chemistry properties, and drug-like characteristics, the top 20 compounds were selected for further analysis. Eleven out of these 20 compounds were purchased for further bioactivity testing (Fig 3A, S9 Fig). Five compounds (**cmpd2, cmpd4, cmpd8, cmpd10, cmpd11**) could inhibit the GTPase activity of UreG by 37% to 72%; subsequent urease assays showed that two compounds (**cmpd4, cmpd8**) exhibited very good activity (by ca. 60% and 90%, respectively) towards inhibition of urease activity in *H*. *pylori* cells (S10 Fig). We then carried out further validation with **cmpd4** and **cmpd8** by using both GTPase and urease assay. The inhibitory concentration (IC)_50_ values towards inhibition of GTPase activity of *Hp*UreG were determined to be 16.8 ± 6.5 and 13.2 ± 7.6 μM for **cmpd4** and **cmpd8**, respectively (Fig 3B), while urease activity in a clinically isolated *H*. *pylori* strain was markedly reduced upon treatment with **cmpd4** and **cmpd8** (Fig 3C) with IC_50(urease)_ of 16.7 ± 6.5 and 9.5 ± 7.6 μM, respectively. For comparison, the IC_50(urease)_ of a clinically used urease inhibitor, AHA, was also determined to be 1321 ± 291 μM. Clearly, both **cmpd4** and **cmpd8** exhibited more potent inhibition towards *H*. *pylori* urease than AHA.

To further evaluate the inhibitory effects of these two compounds on bacterial growth, the compounds were supplemented into the culture medium of *H*. *pylori*, and the pH of brucella broth medium was then adjusted to 5.0 with sterile dilute hydrochloric acid (HCl); inoculum was added to an initial optical density at 600 nm (OD_600_) of ca. 0.1. After 24 h of culture, OD_600_ values were recorded. As shown in Fig 3D, in the absence of inhibitors, *H*. *pylori* exhibited excellent growth in the acidic brucella (OD_600_ = 0.93) compared with that in neutral medium (OD_600_ = 0.18), consistent with a previous report that *H*. *pylori* exhibits facultative acidophilism [35]. In the neutral medium, almost no perturbation on the growth of *H*. *pylori* was found for AHA. However, in the acidic medium, the OD_600_ value of *H*. *pylori* culture was reduced moderately by AHA from 0.93 to ca. 0.71. Evident decreases in the OD_600_ values of *H*. *pylori* culture to 0.23, 0.46, and 0.47 were noted upon the supplementation of CBS, **cmpd4**, and **cmpd8** into the medium, respectively, indicating UreG inhibitors (CBS, **cmpd4**, and **cmpd8**) hindered the growth of *H*. *pylori via* urease inhibition.

### cmpd4 and cmpd8 bind to UreG

**Fig 4:**
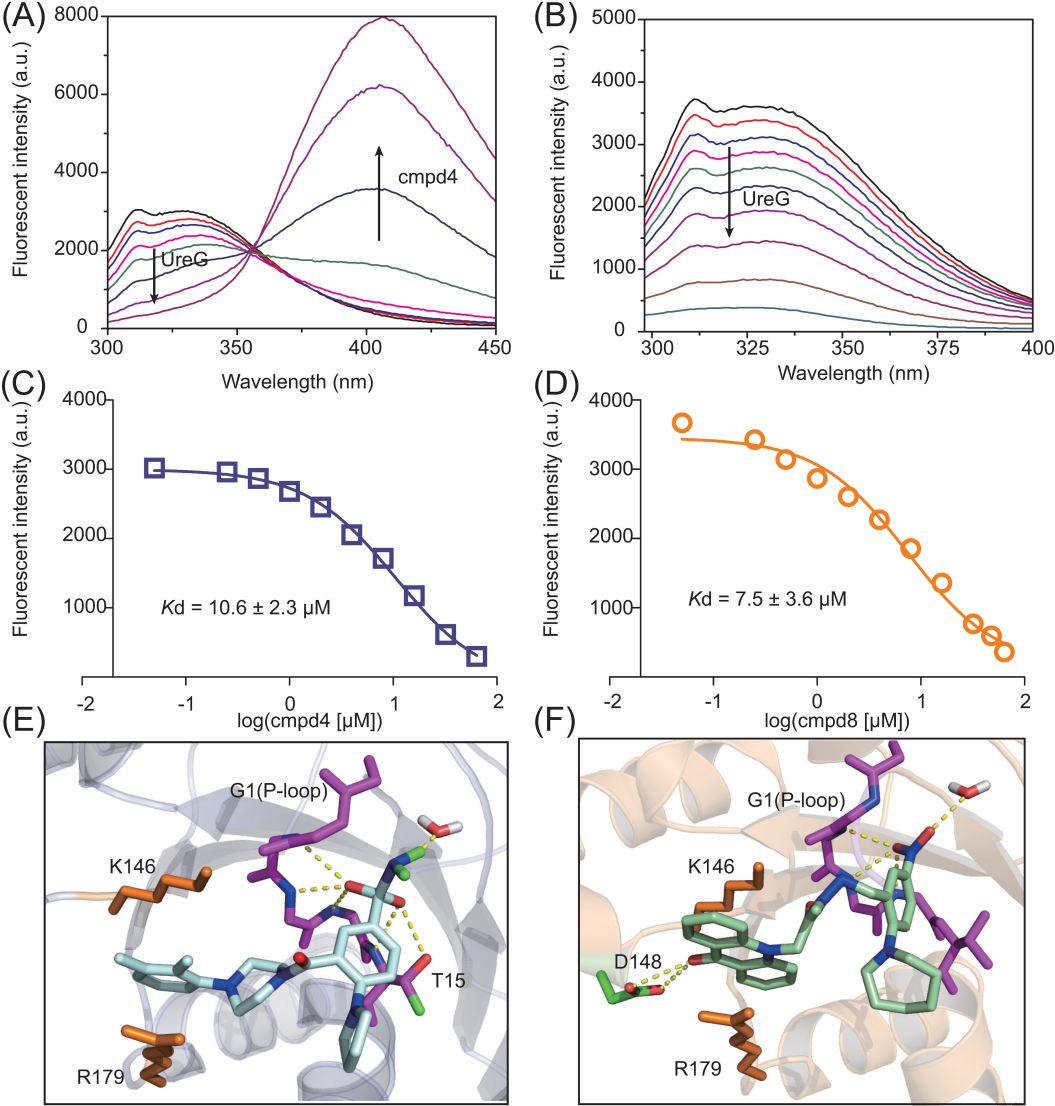
Binding of cmpd4/cmpd8 to UreG by fluorescence measurement and docking models. (A) Fluorescence spectra of UreG upon addition of **cmpd4**. UreG displays fluorescence at 310 nm and 335 nm in the absence of **cmpd4**; binding of **cmpd4** to UreG induced the fluorescence quenching of UreG. (B) Fluorescence spectra of UreG upon addition of **cmpd8**. (C)(D) The representative binding curves of **cmpd4** and **cmpd8** to UreG. (E)(F) **cmpd4** and **cmpd8** bind to the G1(P-loop) motif (magentas) and G4 (NKXD) motif in guanine nucleotide binding pocket of UreG. The underlying data for Fig 4C and 4D can be found in S1 Data.

To investigate whether both **cmpd4** and **cmpd8** bind UreG, we applied a thermal shift assay. As shown in S11 Fig, in comparison to **cmpd1**, which is inactive to inhibit UreG activity, **cmpd4** and **cmpd8** induced thermal shifts (approximately 2.5°C) on the melting temperature of UreG, indicative of binding between the compounds and UreG. Given that UreG itself displays a fluorescent signal at around 310 nm and 335 nm upon excitation at 280 nm, which corresponds to emission of intrinsic Tyr and Trp of UreG, respectively, we further characterized the binding of the compounds to UreG by measurement of the quenching of the intrinsic fluorescence of UreG upon addition of the compounds. The addition of **cmpd4** and **cmpd8** showed negligible effect on the absorption of incident radiation of UreG at 280 nm (S12 Fig), whereas with the addition of **cmpd4** and **cmpd8**, the peaks at 310 nm and 335 nm (corresponding to emission of UreG) were reduced gradually (Fig 4A and 4B); binding affinities (*K*_d_) of UreG to **cmpd4** and **cmpd8** were determined to be 10.6 ± 2.3 and 7.5 ± 3.6 μM, respectively, by nonlinear fitting the curve of fluorescent changes at 310 nm (Fig 4C and 4D). **cmpd4** is a self-fluorescent molecule with its excitations at 250 nm, 280 nm, and 310 nm, and emission at 410 nm (S13 Fig); upon addition of **cmpd4**, the emission peak of 410 nm was elevated (Fig 4A). Interestingly, as one of the excitations of **cmpd4** (310 nm) overlapped with the emission peak of UreG, the presence of a fluorescence resonance energy transfer (FRET) between UreG and **cmpd4** was observed (S14 Fig). For comparison, we also determined the binding affinity of substrate GTP to UreG to be around 65 μM (S15 Fig), indicating that **cmpd4** and **cmpd8** exhibited higher affinity towards UreG than GTP.

Computational models were built to reveal the potential binding interfaces between **cmpd4**/**cmpd8** and UreG (Fig 4E and 4F). The binding sites of small molecules were located at the guanine nucleotide binding pocket (S16 Fig), involving G1 motif (P-loop, magenta color) and G4 motif (NKXD motif). The main chain amide groups of G1 motif wrapped around both **cmpd4** and **cmpd8** (Fig 4E and 4F). For **cmpd4**, the side chain of T15 in G1 motif formed a hydrogen bond with an O atom of **cmpd4**, whereas **cmpd8** was stabilized by the side chains of K146 and D148 in G4 motif. Aliphatic regions of R179 and K146 (red) in G4 motif had extensive hydrophobic interactions with small molecules (Fig 4E and 4F).

To further confirm the interface between UreG and small molecules generated from computational models, we then constructed an UreG mutant (UreGΔNKXD) by replacing selected residues (N145, K146, D148) with Ala to disrupt G4 motif, which abolished GTP binding of UreG (S17 Fig). As shown in S18 Fig, mutation of these amino acids weakened the binding of UreG to **cmpd4** and **cmpd8**, with binding affinities of 34.7 and 33.4 μM. respectively.

We further investigated whether binding of **cmpd4** or **cmpd8** to the guanine nucleotide binding pocket perturbed the Ni(II) coordination to the protein. Considering that coordination of Ni(II) to UreG results in a (Cys)→Ni(II) LMCT band centered at about 337 nm [17], we monitored the UV absorption of UreG samples (5 μM) at 337 nm upon titration of nickel with or without the supplement of **cmpd4** or **cmpd8**. As shown in S19 Fig, **cmpd4** at a ratio of [cmpd4]/[UreG] of 5 and 10 reduced the percentages of Ni(II)-bound UreG to ca. 75% and 30%, respectively, and a similar phenomenon was also observed for **cmpd8** (S19 Fig), indicative of the disruption of nickel binding property of UreG through targeting nucleotide binding site.

### cmpd4 and cmpd8 attenuate *H*. *pylori* virulence in a mammalian cell infection model

**Fig 5:**
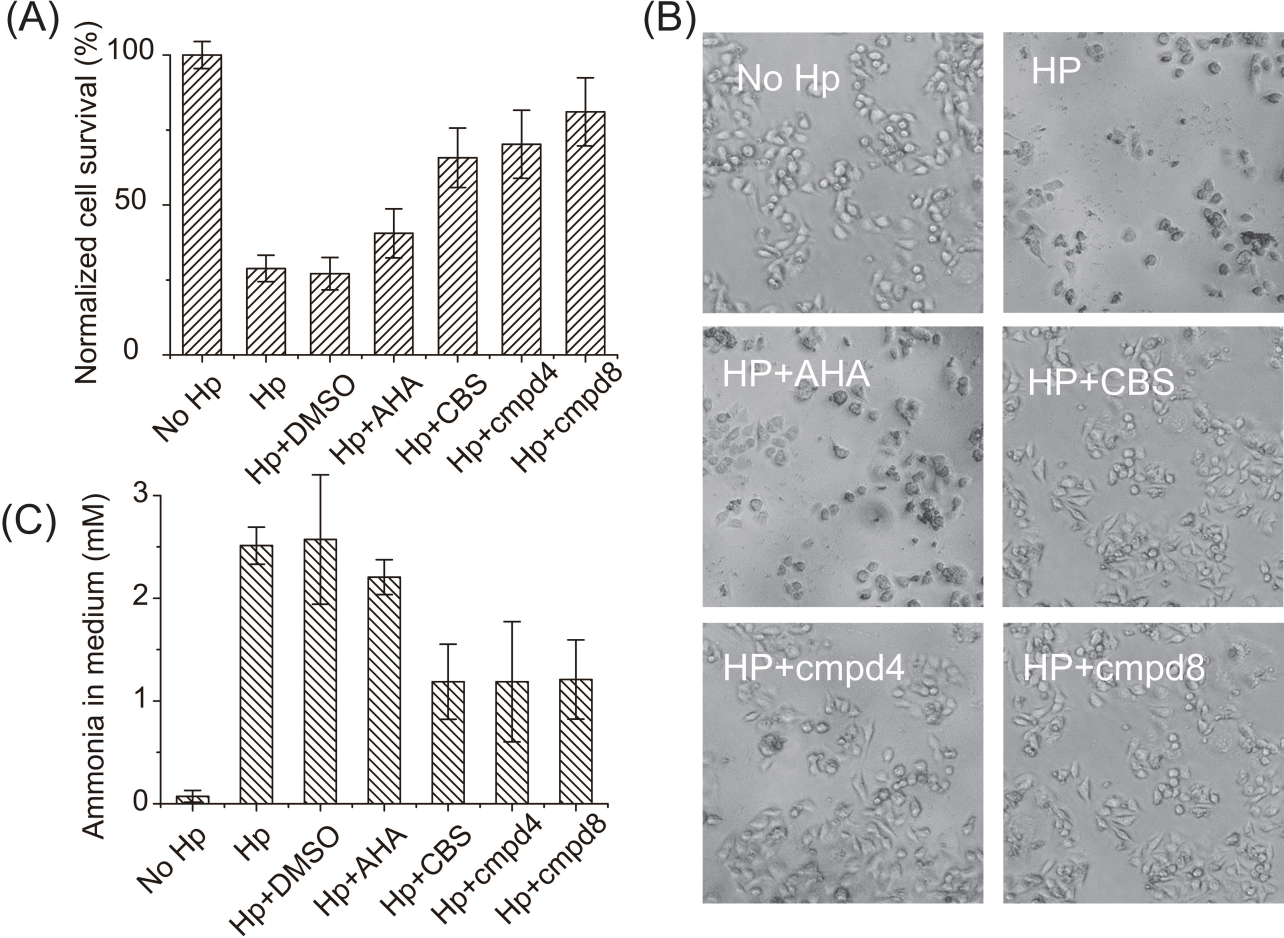
Small compounds attenuate the virulence of *H*. *pylori via* inhibiting urease activity. (A) Viability of gastric cells exposed to *H*. *pylori*. (B) Images of AGS cells after exposure (24 h) to *H*. *pylori* with or without supplementation of AHA, CBS, **cmpd4**, and **cmpd8**. CBS and **cmpd4**/**cmpd8**-treated *H*. *pylori* cells show less toxicity to gastric cells. (C) Amounts of ammonia in cell culture medium after exposure of gastric cell to *H*. *pylori*. Urea was supplied to allow a final concentration of 10 mM. Reduced levels of ammonia are noted in CBS- and **cmpd4**/**cmpd8**-treated groups. The underlying data for Fig 5A and 5C can be found in S1 Data. AHA, acetohydroxamic acid; CBS, colloidal bismuth subcitrate.

We next investigated whether such small molecule inhibitors of urease could be used to treat bacterial infection in a mammalian cell culture infection model. It has been demonstrated that *H*. *pylori* exerts toxic effect on host cells via producing ammonia [36]. AGS, a human gastric adenocarcinoma cell line, was used to evaluate the cytotoxicity of *H*. *pylori* urease to host cells. *H*. *pylori* was cultured microaerophilically in brucella broth medium supplemented with AHA, CBS, cmpd4, or cmpd8 (10 μM), respectively, for 24 h. Harvested *H*. *pylori* cells were normalized according to optical density reading and then cocultured with AGS cells at a multiplicity of infection (MOI) of 20. After 24 h of bacterial infection, the viability of AGS cells exposed to *H*. *pylori* was determined, and the ammonia concentration in medium was measured. As shown in Fig 5, compared to the control group, the survival rate of cultured AGS cells exposed to *H*. *pylori* without small molecule urease inhibitors or with only DMSO in the medium dropped to only 28.8% ± 4.4% and 27.1% ± 5.4% respectively. With the addition of AHA to *H*. *pylori*, viability of AGS cells was determined to be 40.5% ± 8.2%. In contrast, survival rates of AGS cells exposed to *H*. *pylori* with the addition of CBS, **cmpd4**, and **cmpd8** were 65.8% ± 9.9%, 70.3% ± 11.3%, and 81.1% ± 11.3%, respectively (Fig 5A and 5B). Accordingly, the ammonia concentrations in culture medium of the bacteria (HP), bacteria with DMSO treatment (HP + DMSO), and with AHA (HP + AHA) groups were ca. 2.5 mM, which are significantly higher than those groups treated with CBS (HP + CBS) or **cmpd4**/**cmpd8** (HP + cmpd), in which the ammonia concentrations were around 1.1 mM (Fig 5C). All these data imply that these compounds attenuated virulence of *H*. *pylori* during host–pathogen interaction via inhibition of urease activity.

## Discussion

Metalloenzymes serve as potentially druggable targets. Many existing drugs, including acetazolamide and romidepsin, have been designed by targeting zinc metalloenzymes, thus neutralizing their catalytic activity [37]. A common strategy for development of metalloenzyme inhibitors lies in either synthesis of small molecules directly targeting the active sites or identification of substrate mimics that bind to the enzyme with high affinity. However, many metalloenzymes possess complex structures comprising multiple subunits [4,38] with the active sites buried deep inside, which makes it difficult to design enzyme inhibitors for therapeutic purposes. Moreover, the high substrate specificity of many enzymes also hinders the drug development. This is exemplified by bacterial ureases, which have a supramolecular assembly and high substrate specificity [5]. Although urease has long been recognized as a critical virulence factor for microbial pathogenesis [3], there appears to be only one urease inhibitor (AHA) clinically available, and even that has detrimental side effects [39]. Alternative strategies are therefore needed to fully explore the potential of urease as an antimicrobial target. Given that insertion of metal cofactors to the active sites of metalloenzymes, namely enzyme maturation, is a prerequisite for full enzymatic activity, and often involving a series of metallochaperones [40], whereas metallochaperones of bacterial ureases are generally small proteins but essential for assembling the active sites of the enzyme [17,41–43], these metallochaperones might serve as better targets than metalloenzyme itself for designing urease inhibitors.

Bismuth drugs have been used clinically to treat *H*. *pylori* infection since its first discovery; however, the mechanism of action remains obscure [22]. In this report, we intend to understand how bismuth inhibits urease activity in *H*. *pylori* and aim to discover a new target for designing urease inhibitors. By utilizing a homemade fluorescent probe, Bi-*NTA-AC* [27], we show that UreG, which plays a crucial role in urease maturation in microbial pathogens [3], appeared to be the only accessary protein that was found to bind Bi(III) in bacterial cells (Fig 1A). Binding of Bi(III) to UreG led to dissociation of the protein–protein complexes, e.g., UreE-UreG and UreG-UreF-UreH (S3 Fig and Fig 1C), that are important for urease maturation [16,17,31,44]. Consequently, bismuth drugs completely abolished the GTPase activity of UreG (Fig 1D). We further demonstrate that bismuth drugs inactivated urease indirectly by disruption of urease maturation via inhibition of UreG activity (Fig 1E and S5 Fig). This is further confirmed by the observation that CBS could efficiently inhibit urease activity of *H*. *pylori* only in live pathogens, but not for matured urease (Fig 2A). The newly expressed urease in live bacterium is in an apo-form, and binding of CBS to UreG disrupted the delivery of nickel cofactor to the active site of urease. In contrast, inhibition of urease activity in vitro was very inefficient, even at millimolar concentration [24]; this is attributable to the deeply buried active sites of urease, which is less prone to be attacked by inhibitors [4,5]. Our combined data provide solid evidence on the inefficiency of urease inhibitors designed based on directly targeting the active site of urease.

Besides its well-known role in acid acclimation for *H*. *pylori*, urease is also associated with the development of infection stones caused by bacterial urinary tract infections, including those caused by *Klebsiella* and *Proteus* species. Moreover, emerging roles of urease in different processes of microbial infection have also been established, such as participating in nitrogen metabolism [45,46], resisting peroxynitrite pressure [47], avoiding destruction by phagolysosomes [8], and facilitating pathogen crossing of the blood–brain barrier [9]. Apparently, there is a great medical urgency for the development of urease inhibitors to treat urease-related microbial infections, which has not been fully explored. In this study, we further show that inactivation of urease activity via disruption of urease maturation process by bismuth was not specific for *H*. *pylori*. Instead, similar phenomena were also found in several other bacterial pathogens, such as *P*. *mirabilis*, *P*. *aeruginosa*, *K*. *pneumoniae*, *L*. *hongkongensis*, *S*. *aureus*, and *S*. *saprophyticus* (Fig 2C). Thus, it is highly possible that CBS, which is already a clinically used anti-ulcer drug, could be repurposed for the clinical use of treating urease-related microbial infection.

Moreover, considering that UreG is highly conserved among urease-producing microbial species, UreG might serve as a good target for the design of urease inhibitors. Using *H*. *pylori* UreG as a showcase, we performed a virtual screening from 1,700 compounds and tested 11 hits. Our detailed validation shows that two compounds exhibited good inhibition towards GTPase activity of UreG with IC_50_ at μM level (Fig 3B). Moreover, these two compounds exhibited more potent inhibitory effects on urease activity in *H*. *pylori* cells than the clinically used urease inhibitor AHA (Fig 3C). Distinct from CBS, these two small molecules targeted nucleotide binding pockets instead of nickel binding sites of UreG (Fig 4, S16 Fig and S18 Fig). The efficacy of the two lead compounds was further validated both in culture medium of *H*. *pylori* and in a mammalian cell infection model. (Fig 3D, Fig 5). Taken together, we demonstrate that the compounds targeting UreG could indeed inhibit urease activity and therefore possess potent antimicrobial activity.

Hydroxamic acids are the most widely studied group of urease inhibitors [13]. They inhibit urease through attacking the nickel ions in the active site of urease owing to their well-known metal-complexing properties [48]. However, this kind of compounds exerts only moderate inhibitory activity (as shown in S7 Fig, the IC_50_ values of AHA against maturated ureases reach as high as mM level). Although AHA has been introduced into clinical use for urinary tract infections, high doses (approximately 1,000 mg/d for adults) are required due to its relatively low anti-urease activity; thus, severe side effects are noted [13]. Another group of urease inhibitors, which were considered as substrate analogues, shares similar structural characteristics with urea and competes with the substrate binding. Due to high specificity of urea to urease, such inhibitors also only exhibit moderate activity; for example, boric acid acts as a competitive inhibitor of urease from jack bean, *P*. *mirabilis*, and *K*. *aerogenes*, with *K*_i_ values at mM levels [12]. In contrast, CBS, **cmpd4**, and **cmpd8** inactivated urease in bacteria through targeting accessory protein UreG, either at nickel binding site or GTP binding site, exhibiting more potent activity than clinically used AHA (Fig 3C and S7 Fig). Current compound libraries used in this study were designed for mammalian protein targets such as *Homo sapiens* and *Rattus norvegicus* [33]. **cmpd4** and **cmpd8** have also been found to be active in several high throughput screenings (HTSs) against targets in human cells, such as G protein-coupled receptor 55 (GPR55), Interleukin 1 beta (IL1β), Focal Adhesion Kinase (FAK), and Regulator of G-protein Signaling 7 (RGS7) [49–52]. As expected, these compounds might exhibit toxicity to mammalian cells. Indeed, administration of small molecule inhibitors to AGS cells infected by *H*. *pylori* resulted in low viability of AGS (S20 Fig), due to the toxicity of small compounds to mammalian cells. Therefore, it may be necessary to fine-tune structures of **cmpd4** and **cmpd8** to increase their selectivity and potency based on the rationale that the structure and regulation of bacterial GTPase UreG is distinct from mammalian proteins.

Despite the fact that UreG was selected as a showcase study, such a strategy should have a broad application in the development of metalloenzyme inhibitors. Indeed, proteins that are involved in metalation pathways have also been considered as good targets for the development of new classes of pharmaceutical agents [53]. A recent study also demonstrated that small molecules that inhibit the human copper trafficking proteins Atox1 and CCS significantly reduce proliferation of cancer cells, suggesting copper chaperones as new targets for the development of anticancer therapies [54].

In summary, we have discovered a metallochaperone, UreG, as a new target for the design of urease inhibitors based on extensive mechanistic study of bismuth inhibition of urease. Using virtual screening in combination with experimental validation, we demonstrate that compounds that bind and functionally perturb GTPase activity of UreG could indeed inhibit urease activity both in culture medium of *H*. *pylori* and in a mammalian cell infection model. Our study clearly indicates that metallochaperones participating in maturation of important microbial metalloenzymes serve as new targets for devising antimicrobial drugs. In comparison with conventional antibiotics, such antimicrobials might have less likelihood of encountering antimicrobial resistance in pathogens, considering the crucial roles of these metalloenzymes in microbial pathogenesis that are less susceptible to mutation.

## Materials and methods

### Construction of expression plasmids

*ureG* gene was amplified by PCR from the genomic DNA of *H*. *pylori* strain 26,695 using the primer pair UreG-For/UreG-Rev (S1 Table), after which sites of restriction endonucleases *Nde*I and *Eco*RI would be encoded at the 5′- and 3′- end of the PCR product, respectively. The PCR product and the cloning vector pET28a were digested with the restriction endonucleases (New England Biolabs), followed by T4 ligation using T4 DNA ligase (New England Biolabs) to produce the expression plasmid pET28a-UreG. For generating the UreG variants UreG-C48A, UreG-C66A, UreG-C48C66A, and UreGΔNKXD, pET28a-UreG served as the template for site-directed mutagenesis using the Phusion High-Fidelity PCR Kit (New England Biolabs).

For analysis of the effect of Bi-binding to UreG on the GTPase activity of UreG and the urease maturation, plasmid pHP8080 was used as the template for the generation of a plasmid with the urease gene cluster excluding the *ureG* gene (i.e., *ureG* gene-deleted plasmid named pHP8080ΔG) using primer pair UreA2HΔG-For/UreA2HΔG-Rev.

### Identification of Bi(III)-binding proteins in *H*. *pylori* by Bi-*NTA-AC*

To identify the Bi(III)-binding proteins in live *H*. *pylori* cells, Bi-*NTA-AC* was applied to the bacterium according to previous reports [27,28]. Prior to treatment with Bi-*NTA-AC*, the bacterial cells were collected by centrifugation at 4,000 g at 4°C for 5 min and were washed in PBS (pH 7.4) three times. Cell pellets were resuspended in PBS (pH 7.4), and cells were then incubated with Bi-*NTA-AC* (50 μM) at 37°C for 30 min with agitation. After that, cells were washed with PBS (pH 7.4) three times prior to exposure to UVP UVGL-25 Mineralight UV lamp for 10 min to allow the formation of covalent linkage between the probe and the labeled proteins.

Cells (with or without Bi-*NTA-AC* treatment) were lysed by sonication in PBS buffer supplemented with 1 mM PMSF, after which the supernatant was obtained by centrifugation at 10,000 g for 30 min at 4°C. The protein concentration in the cell lysates was estimated using BCA protein assay (Novagen).

The cell lysates were subjected to analysis by 2-dimensional gel electrophoresis (2-DE). The samples were desalted by 2-D Clean-Up Kit (GE Healthcare), while the proteins precipitated were rehydrated by rehydration buffer (8 M Urea, 2% [w/v] CHAPS, 0.28% [w/v] DTT, 2% [v/v] IPG buffer 3–10 NL, and 0.002% [v/v] bromophenol blue). Isoelectric focusing was performed by Immobiline DryStrip pH 3–10, 13 cm (GE Healthcare) at room temperature. The strips were actively rehydrated for 12 h at 30 V, followed by steps of salt removal: 500 V for four hours and 1,000 V for 1 h. The voltage was then gradually increased to 8,000 V in 2 h and was maintained constant at 8,000 V for an additional 7 h.

The proteins were further analyzed by 13% SDS-PAGE. The IEF strips were first treated with DTT (10 mg/mL in equilibrating buffer [6 M Urea, 75 mM Tris, pH 8.8, 2% SDS, 30% glycerol, and 0.002% bromophenol blue]) for 15 min at room temperature and were subsequently treated with iodoacetamide (25 mg/mL in the same equilibrating buffer). The IEF strips were sealed onto SDS-PAGE with agarose sealing solution (0.5% agarose in SDS-PAGE running buffer with 0.002% bromophenol blue), after which the SDS-PAGE was resolved at 30 mA/gel for 5 h. The gel (with Bi-*NTA-AC* treatment) was washed in distilled water for 1 h prior to fluorescent imaging on ImageQuant 350 (GE Healthcare) at an excitation wavelength (λ_ex_ = 365 nm). The gels were subsequently subjected to silver staining.

The protein spots after fluorescence staining and silver staining were compared, while the identities of the proteins were confirmed by MALDI-TOF MS to identify the potential Bi-binding proteins in *H*. *pylori*.

### Bacterial culture

*P*. *mirabilis*, *P*. *aeruginosa*, *K*. *pneumoniae*, *L*. *hongkongensis*, *S*. *aureus*, and *S*. *saprophyticus* were cultured in LB broth overnight at 37°C. The overnight culture was inoculated into fresh LB medium at OD_600_ of approximately 0.4; CBS (0, 2, 5, 10, 20, 50, 100, 200, 400 μM) was supplemented into the culture medium. *H*. *pylori* strain 26,695 or a clinical-isolated strain were cultured on Columbia agar base in the presence of 7% laked horse blood (Oxoid) and *H*. *pylori* selective supplement Dent (Oxoid) at 37°C under microaerobic conditions using Campygen 2.5L (Oxoid) for 3 d. The bacteria were then inoculated at OD_600_ of approximately 0.4 to Brucella broth in the presence of 1.4% β-cyclodextrin overnight at 37°C with agitation with the supplement of CBS (0, 2, 5, 10, 20 μM) into the culture medium.

### Protein purification

Expression plasmids (pET28a-UreG, pET28a-UreG-C48A, pET28a-UreG-C66A, pET28a-UreG-C48C66A, and pET28a-UreGΔNKXD) were transformed into *E*. *coli* BL21(DE3). An overnight culture was then inoculated into fresh LB broth containing 50 μg/mL kanamycin and was incubated at 37°C. Protein expression was induced at the OD_600_ of 0.6 using 0.2 mM IPTG and was carried out at 25°C overnight. Centrifugation was done to collect cells.

Cells were lyzed by sonication in Tris buffer (20 mM Tris-HCl, pH 7.4, 500 mM NaCl, and 20 mM imidazole) in the presence of 1 mM PMSF, after which centrifugation of cell lysate at 10,000 g at 4°C for 30 min was done to remove the inclusion body. Supernatant was filtered through 0.45-μm filter unit (Sartorius) and was then applied to HisTrap Ni-NTA column (GE Healthcare) pre-equilibrated with the same Tris buffer. Proteins were washed and were eluted with the same Tris buffer containing 50 mM and 300 mM imidazole, respectively.

The fraction containing His-tagged UreG (or its variants) was buffer-exchanged into a Tris buffer of lower salt concentration (20 mM Tris-HCl, pH 7.4, 120 mM NaCl] for cleavage of the His-tag by Thrombin (Sigma) at 20°C overnight, followed by the removal of uncleaved proteins using HisTrap Ni-NTA column. The cleavage product, UreG (or its variants), was treated with 5 mM EDTA overnight at 4°C to obtain the apo-form of the proteins.

UreG (or its variants) was further subjected to purification using HiLoad 16/10 Superdex 75 size exclusion column (GE Healthcare) in HEPES buffer (20 mM HEPES, 300 mM NaCl, pH 7.4) with the supplement of 500 μM TCEP for reducing the disulphide bonds in the protein. The peak fractions were concentrated, whereas the protein concentration was estimated by BCA Protein Assay Kit (Novagen). The metal contents in the protein samples were analyzed by ICP-MS to ensure the proteins were not bound with any metal ions prior to other experiments.

### UV-vis spectroscopic analysis

To monitor binding of Bi(III) ions to proteins, UV-vis spectroscopy was employed. The spectra were recorded at room temperature from 500 to 280 nm at a scan speed of 240 nm/min using a 1-cm quartz cuvette. Protein samples (25 μM) were prepared in HEPES buffer (20 mM HEPES, 100 mM NaCl, pH 7.4) with 500 μM TCEP, while various concentrations of Bi(III) ions (using BiNTA as the Bi(III) source) were titrated into apo-UreG or its variants (UreG-C48A, UreG-C66A, and UreG-C48C66A). The solution was allowed to incubate for 10 min prior to recording of the UV spectra. The dissociation constant (*K*_d_) of BiNTA to UreG was estimated by nonlinear fitting of the titration curve using the Ryan–Weber equation below.

$$\Delta F=C\frac{([P]+[L]+K_{d})-\sqrt{{\left([P]+[L]+K_{d}\right)}^{2}-4[P][L]}}{2}$$

where Δ*F* refers to the change in signals (absorbance), *C* is the parameter for the change in signals per unit complex, [*P*] refers to the protein concentration, and [*L*] is the concentration of metal ions. The dissociation constant (*K*_d_) of BiNTA binding to UreG was estimated to be 1.1 μM using the Ryan–Weber equation. Given that log *K*_a_ of Bi-NTA is 17.55 [55], the apparent binding constant of Bi(III) ions to UreG was calculated to be *K*_d_/*K*_a_ = 3.1 × 10^−24^ M.

To study the interplay between Bi(III) and Ni(II) towards binding to UreG, similar UV-vis spectroscopic analysis was performed under different conditions. To investigate whether Bi(III) ions affect the Ni(II)-binding ability of UreG or vice versa, Ni(II)- and Bi(III)-bound UreG was first prepared by incubating UreG protein with two molar equivalents of either Ni(II) ions (as NiSO_4_) in HEPES buffer supplemented with 500 μM TCEP, 100 μM GTP, and 1 mM MgSO_4_ at 4°C for 1 h or Bi(III) as (BiNTA). Bi(III) or Ni(II) ions (0 to 3 molar equivalents) were subsequently titrated into Ni- or Bi-bound UreG, respectively, prior to recording UV spectra. In order to further examine the mode of interaction of Bi(III) ions with UreG, Bi(III) ions were titrated into Ni-bound UreG in the presence of 1 mM KHCO_3_ and two molar equivalents of GTP or GTPγS (a nonhydrolyzable analogue of GTP, guanosine 5'-O-(3-thiotriphosphate).

### Analysis of oligomeric state of UreG and protein-protein interactions by analytical size exclusion chromatography

Oligomerization states of UreG, UreE-UreG (2E-2G complex), and UreG-UreFH (2G-2F-2H complex) upon different treatment were studied by chromatography using Superdex 200 10/300 GL column (GE Healthcare), which was calibrated with the Gel Filtration Calibration Kit (low molecular weight) (GE Healthcare). Ni-loaded UreG was prepared by mixing UreG with NiSO_4_ (1x), GTP (10x), and MgSO_4_(1 mM). The UreG-UreFH (2G-2F-2H complex) was prepared by mixing the apo-UreFH complex with excess apo-UreG. UreE-UreG (2E-2G complex) was prepared by mixing apo-UreE and apo-UreG in the presence of GTP and MgSO_4_. Ni-loaded UreG, 2E-2G, and 2G-2F-2H were incubated with two molar equivalents of Bi(III) ions (as BiNTA) prior to analysis with analytical size exclusion chromatography.

### Virtual screening for UreG-binding small molecules

A virtual screening method was used to find the potential inhibitors of UreG. A set of 1,700 compounds was collected from the PubChem [33] and BindingDB [34] databases for screening using AutoDock Vina [32]. A total of 300 compounds are active from PubChem Bioassay (AID No.: 588479, 588622, 759) that already detected as GTPase inhibitors [56–58]. Also, 1,400 compounds have been extracted from BindingDB by searching “G-protein” term as target name in advanced search mode. Chemoinformatics python library RDKit [59] was used in python 2.7 to generate 3D conformation of these compounds using universal force field (UFF). In the next step, python script “prepare_ligand4.py,” which is developed by AutoDockTools [60], was used to convert the small molecules structures to PDBQT format, which is a required format in AutoDock Vina’s virtual screening procedure.

*H*. *pylori* urease accessory protein UreG complex (PDB 4HI0) was used as the docking receptor. Using MGLTool, the PDB structure of the receptor was converted to PDBQT format. A binding box with 22 × 16 × 15 Å^3^ dimensions (1 Å step size) with center point (−48.231, −0.779, 50.178) defined for 4HI0. Finally, a python script was developed to run AutoDock Vina on HKU high-performance computing (HPC) center, the compounds have been ranked based on their docking scores with 4HI0, and the best 20 compounds have been selected for further analysis. Eleven out of these 20 compounds were commercially available in MolPort and were purchased for further bioactivity testing. The chemical structure information of the tested compounds is available in the PubChem Substance through the substance identifier numbers (SIDs) as follows: cmpd1:SID = 88333436 [61], cmpd2:SID = 97342323 [62], cmpd3:SID = 97368460 [63], cmpd4:SID = 91955059 [64], cmpd5:SID = 93231017 [65], cmpd6:SID = 88183171 [66], cmpd7:SID = 88329940 [67], cmpd8:SID = 89225247 [68], cmpd9:SID = 123792418 [69], cmpd10:SID = 88426485 [70], cmpd11:SID = 88662278 [71].

### *K*_d_ measurements for cmpd4, cmpd8 and GTP-binding of UreG

To calculate the *K*_d_ values, the fluorescence-quenching titrations were carried out; gradient amounts of **cmpd4**, **cmpd8**, and GTP (0–100 μM, less than 20 μl DMSO in 1 ml buffer) were titrated into 1 μM UreG in assay buffer. Using the relation between the emission intensity at 310 nm and molecule-UreG–bound species, as well as one site-binding model, the *K*_d_ values were calculated by a logistic regression model referring to a previous report [54]. The experiments were carried out in 20 mM HEPES, 100 mM NaCl (pH 7.4). All fluorescence readings were corrected for the dilution effect.

### Measurement of GTPase activity of UreG

A Malachite Green Phosphate Assay Kit (Abcam) was utilized to quantify the hydrolysis of GTP by UreG under various conditions. Ni-bound UreG was incubated with different amounts of CBS or small compounds in GTPase assay buffer (20 mM HEPES, 100 mM NaCl, pH 7.4, 1 mM MgCl_2_, 1% glycerol, 50 μM GTP) at 4°C for 30 min. GTPase activity of UreG was triggered through the addition of 10 mM KHCO_3_, after which the reaction was incubated at 37°C for 30 min; then, the free phosphate from hydrolysis of GTP was determined. Control experiments were conducted to ensure that Bi(III) does not interfere with the malachite green assay. To eliminate the potential effect of small compounds on malachite green assay, the assay included a subtraction of the absorbance of reaction with the same amounts of compounds but without UreG enzyme for each reading. The IC_50_ values for CBS or small compounds against GTPase activity were calculated using a logistic regression model. For comparison, the GTPase activity of negative control (reaction without UreG enzyme, in the absence of GTPase inhibitors) was set as 0, and the activity of positive control (reaction with Ni-bound UreG, in the absence of GTPase inhibitors) was set as 100% (as shown in Fig 1D, Fig 3B, S5 Fig, and S10 Fig).

### Phenol–hypochlorite urease assay

Phenol–hypochlorite urease assay was performed to examine the activity of urease in live bacteria or extracted urease and to investigate the role of UreG on the urease activity. Control experiments were conducted to ensure no interference from Bi(III) and small compounds on phenol–hypochlorite urease assay.

To understand the effect of Bi(III) binding to UreG on urease maturation, plasmid pHP8080ΔG was transformed into *E*. *coli* BL21(DE3), and the overnight bacterial culture was inoculated into fresh LB broth for growth at 37°C until OD_600_ was about 0.6. Cells were washed three times with M9 minimal medium. Protein expression was carried out in M9 minimal medium in the presence of 0.2 mM IPTG. Cells were harvested by centrifugation and subsequently washed with HEPES buffer (50 mM HEPES, pH 7.5). The cell pellet was resuspended in the same HEPES buffer, which was then lyzed by sonication. Supernatant was obtained by centrifugation at 15,000 g at 4°C for 5 min, while BCA Protein Assay Kit was employed to estimate the protein concentration in the supernatant. Sequentially, purified UreG protein (with or without the treatment with metal ions) was supplemented to the cell lysate. Ni-bound UreG was prepared by incubating UreG protein with two molar equivalents of Ni(II) ions (as NiSO_4_), 10 molar equivalents of GTP, and 1 mM MgSO_4_ at 4°C for 1 h in HEPES buffer supplemented with 500 μM TCEP. To study the effect of CBS on the activity of UreG, Ni-bound UreG was incubated with CBS (0–3 molar equivalents) at 4°C for 30 min and then added to the cell lysate. Cell lysate with the addition of apo-UreG and excess Ni(II) ions were also prepared to serve as control.

Treatment of cell lysate (40 μL) under various conditions was done prior to mixing with 250 μL of urease buffer (50 mM HEPES, 25 mM urea, pH 7.5), followed by incubation at 37°C for 30 min. Urease reaction was stopped by the addition of 375 μL of reagent A (10 g/L phenol, 50 mg/L sodium nitroprusside), followed by the addition of 375 μL of reagent B (5 mg/mL sodium hydroxide, 0.044% [v/v] sodium hypochlorite). The samples were further incubated at 37°C for 30 min prior to measurement of the absorbance at 620 nm using a microplate reader. A series of NH_4_^+^ standard (from NH_4_Cl) was also prepared for calibration. Results were normalized against the urease activity measured in the presence of Ni-UreG for comparison.

For investigating the direct effect of CBS and AHA on urease enzymes of different bacterial species, ureases of different bacterial species were extracted. Various concentrations of CBS or AHA were then added to the extracted ureases, after which the urease activity was determined. The IC50_(urease)_ values for AHA and CBS were calculated using a logistic regression model.

Alternatively, for the analysis of urease activity in live bacteria (with and without supplementation of CBS or small compounds in cultured medium), bacteria were collected by centrifugation at 4,000 g for 5 min at 4°C and then lyzed by sonication. Cell lysate were briefly centrifuged at 16,000 g for 5 min at 4°C to remove the inclusion body. The supernatant of different bacterial species was subjected to urease assay analysis. Typically, the urease activity of negative control (reaction without urease enzyme) was set as 0, and the activity of positive control (urease without treatment of potential urease inhibitor) was set as 100%, unless defined otherwise (Fig 1E, Fig 2, Fig 3C, S5 Fig and S7 Fig).

### Cell infection model

AGS cells were grown in 24-well Falcon plastic tissue culture dishes to approximately 80% confluency. *H*. *pylori* was cultured in brucella broth medium in the presence of AHA, CBS, or small molecule urease inhibitors (**cmpd4**, **cmpd8**) at concentrations below IC_50_ values for 24 h microaerophilically and was added to the AGS cells on 24-well plastic tissue culture dishes at a final concentration of 5 × 10^6^ CFU/ml. Urea was added to allow a final concentration of 10 mM. After further 24 h of culture, cell viability of AGS cells exposed to *H*. *pylori* was determined by cell counting, and the ammonia concentration in medium was measured.

### Thermal shift assay

The binding of **cmpd4** and **cmpd8** to UreG was confirmed by a thermal shift assay. The melting points of UreG in the absence of or in the presence of small compounds (50 μM) were measured using Protein Thermal Shift Dye Kit (Thermo Fisher SCIENTIFIC) according to the manufacturer’s protocol.

## Supporting information

S1 Data (XLSX)

S1 Table List of primers for plasmid construction. The mutation sites are indicated in red.
(DOCX)

S1 Fig UV-vis spectra of proteins with or without addition of BiNTA. (A) UreE, (B) UreFH complex, (C) UreG-C48A, (D) UreG-C66A, and (E) UreG-C48C66A. The peaks at approximately 340 nm and approximately 360 nm indicated Bi(III) binding to Cys66 and Cys48 (in UreG-C48A and UreG-C66A), respectively (C, D). the shift of absorption peaks may be due to the different coordination or circumstances.
(PNG)

S2 Fig Interplay between Bi(III) and Ni(II) binding to UreG. Given the critical role of GTP and Mg(II) in Ni-binding of UreG, UV spectroscopic studies were carried out in HEPES buffer containing 100 μM GTP and 1 mM MgSO_4_. (A) UV spectra of Bi-UreG upon addition of zero to two molar equivalents of Ni(II) ions. (B) UV spectra of Ni-UreG upon incubation with up to three molar equivalents of Bi(III) ions. It is noted that addition of Bi(III) to Ni-UreG did not suppress the characteristic peak at approximately 337 nm (π(S)(Cys)→Ni(II) LMCT), while the LMCT peak of π(S)(Cys)→Bi(III) (approximately 350 nm) remained undetectable, indicating the lack of Bi(III) coordination to UreG protein when the metal binding site is preloaded with Ni(II). (C) UV spectra of Ni-UreG upon incubation with up to three molar equivalents of Bi(III) ions in the presence of GTPase-activating element KHCO_3_ (1 mM). Gradual addition of Bi(III) to UreG solution led to a decrease in intensity of the peak at approximately 337 nm and the emergence of a peak at approximately 350 nm, indicative of the simultaneous replacement of Ni(II) ions by Bi(III) on UreG protein. (D) UV spectra of Ni-UreG(GTPγs) upon incubation with up to three molar equivalents of Bi(III) ions in the presence of KHCO_3_ (1 mM). The characteristic Ni-binding peak was not disturbed, while the typical Bi coordination peak was unnoticeable even after the supplementation of excess Bi(III). It is noted that Bi(III) only disturbs UreG dimer at its GTPase transition state (i.e., in the presence of GTPase-activating elements), but not at its stable Ni, GTP-bound state.
(PNG)

S3 Fig Effect of Bi(III) on UreG dimer and UreE-UreG complexes by gel filtration chromatography. (A) Oligomeric states of Ni-UreG with (red curve) or without (black curve) two molar equivalents of Bi(III) treatment in the presence of KHCO_3_ (1 mM). (B) Oligomeric states of UreE-UreG complex (2E-2G) with (red curve) or without (green curve) molar equivalents of Bi(III) treatment.
(PNG)

S4 Fig Gel filtration profiles of UreE with or without Bi(III). Apo-UreE was eluted at approximately 13.5 ml corresponding to its dimeric form. Incubation with three molar equivalents of Bi(III) has little effect on the UreE dimer.
(PNG)

S5 Fig Normalized urease and GTPase activity of *E*. *coli* cells expressing the completed urease gene. The *ureG* gene (plasmid pET32a-*ureG*) was complemented to *E*. *coli* cells harboring plasmid pHP8080ΔG; the expression of ureG gene was induced by 100 μM IPTG. After growth, with the addition of gradient amounts of CBS in cultured medium, the GTPase and ureolytic activities of *E*. *coli* cell lysate were monitored simultaneously. As UreG was overexpressed, the GTPase activity of *E*. *coli* cell lysate was associated with UreG. For convenient comparison, the activities of enzymes (GTPase and urease) in the samples without CBS treatment were set as 100%; the activities of the negative control (without addition of cell lysate into the reactions) were set as 0. The underlying data can be found in S1 Data.
(PNG)

S6 Fig Ni content of *H*. *pylori* cells with the addition of Bi as CBS in cultured medium. *H*. *pylori* was cultured with or without supplementation of Bi(III) to medium. After harvest and washing, the Ni content of *H*. *pylori* cells was determined by ICP-MS sequentially. For convenient comparison, the Ni contents in the samples without CBS treatment were set as 100%. As *H*. *pylori* has an efficient system for nickel sequestration, *H*. *pylori* was cultured without supplementation of excess Ni(II) in cultured medium. The underlying data can be found in S1 Data.
(PNG)

S7 Fig Comparison of inhibition of urease by CBS and AHA in different bacteria. AHA exerts only moderate inhibitory activity against urease with IC_50_ values at around mM levels, whereas CBS exhibits more potent efficiency on anti-urease activity in bacteria cells. For convenient comparison, the activities of urease in the samples without CBS/AHA treatment was set as 100%; the activities of the negative control (without addition of cell lysate into the reactions) were set as 0. The underlying data can be found in S1 Data.
(PNG)

S8 Fig Nickel-dependent GTPase UreG is conserved in various bacteria. Chaperone UreI, which is not required for urease maturation, has not been illustrated in the figure.
(PNG)

S9 Fig The structures of the 11 representative hits from the virtual screening (cmpd1–cmpd11). (PNG)

S10 Fig GTPase assay and urease assay for validation of compounds from virtual screening. (A) GTPase activity of purified Ni-UreG (5 μM) in the presence of 20 μM small compounds. cmpd7 resulted in serious precipitation in the reaction, which led to the high absorption at 620 nm and false high activity. (B) urease activity of *H*. *pylori* cells with the supplement of 20 μM small compounds in cultrued medium. Five tested compounds (cmpd2, cmpd4, cmpd8, cmpd10, and cmpd11) exhibited inhibition effect on the GTPase activity of UreG, whereas cmpd4 and cmpd8 exhibit relatively good anti-urease activity. The underlying data can be found in S1 Data.
(PNG)

S11 Fig Thermal shift assay to confirm binding of cmpd4 and cmpd8 to UreG. cmpd1, an inactive molecule, was used as a negative control. The underlying data can be found in S1 Data.
(PNG)

S12 Fig **UV-vis spectra of UreG with cmpd4 (A), and cmpd8 (B).** Both cmpd4 and cmpd8 (100 μM) alone or in the presence of UreG (1 μM) gave rise to intense absorbance at around 280 nm, whereas the spectra of cmpd4/8-UreG mixture obtained after subtracting the spectra of cmpd4/8 are similar to those of UreG, indicating the presence of cmpd4/8 has negligible effect on the absorption of incident radiation of UreG at 280 nm.
(PNG)

S13 Fig **Excitation (blue) and emission (red) spectra of cmpd4 (A) and cmpd8 (B).** cmpd4 (100 μM) showed λ_ex_ = 250, 280, 310 nm and λ_em_ = 410 nm, whereas the excitation maxium of cmpd8 (20 μM) was observed at 257 nm and emission maximum at 440 nm. cmpd8 exhibited little excitation at 280 nm; thus, only UreG and cmpd4 have significant fluorescent signals with λ_ex_ = 280 nm.
(PNG)

S14 Fig **(A) Fluorescence spectra of cmpd4 (B) Titration of cmpd4 produced FRET between UreG and cmpd4.** When the mixture of UreG and cmpd4 was excited at 280 nm, the emission intensity at 410 nm (FL_UreG+cmpd4_, as shown in Fig 4) consisted of two components: the direct emission of cmpd4 and the emission of cmpd4 excited by energy transferred from UreG. Therefore, the FRET emission of cmpd4 (Em_FRET_) can be determined by Em_FRET_ = FL_UreG+cmpd4_ − FL_cmpd4_.
(PNG)

S15 Fig Representative binding curve of GTP to UreG protein. The dissociation constant of GTP to apo-UreG was determined to be ca. 65 μM, consitent with the previous report that apo-UreG does poorly at GTP-binding. The underlying data can be found in S1 Data.
(PNG)

S16 Fig Surface representation showing guanine nucleotide binding pocket of UreG. (A) GDP (PDB: 2HI0), (B) cmpd4, and (C) cmpd8. The G1 (P-loop) motif is in magenta, and residues K146 and R179 of UreG are in red, which provide potential hydrophobic interaction with compounds.
(PNG)

S17 Fig Gel filtration profiles of protein UreGΔNKXD with or without GTPγs. UreG recognizes GTP using the canonical NKXD motif (G4) and is likely to form UreG dimer upon Ni and GTP binding. To examine the effect of the triple mutagenesis (N145A/K146A/D148A) on GTP binding of UreG, UreGΔNKXD (10 μM) was incubated with or without GTPγs (30 μM) in HEPES buffer (20 mM HEPES, 100 mM NaCl, 30 μM NiSO_4_, 5 mM MgSO_4_, pH 7.4) and was subjected to gel filtration chromatography. Both UreGΔNKXD samples (with or without GTPγs) were eluted as monomers at approximately 14 ml, and the intensity of peaks at approximately 18 ml corresponding to GTPγs in the presence of UreGΔNKXD remained unchanged compared with that of GTPγs alone, implying that UreGΔNKXD has no GTP binding ability.
(PNG)

S18 Fig Representative binding curves of cmpd4 and cmpd8 to UreGΔNKXD. The mutant exhibits lower binding affinity (3 to 4 folds) towards cmpd4/cmpd8 compared to the wild-type UreG. The underlying data can be found in S1 Data.
(PNG)

S19 Fig Effect of cmpd4 and cmpd8 on nickel binding property of UreG. UreG (5 μM) in HEPES buffer (5 μM GTP, 1 mM MgSO_4_) was titrated with NiSO_4_ in the presence of **cmpd4** or **cmpd8** at various concentrations. Titration of nickel ion into UreG samples led to the increasing of absorption at 337 nm, the highest of which was set as 100% for the percentage of Ni-bound UreG. The supplement of **cmpd4** and **cmpd8** reduced the percentage of Ni-bound UreG. The underlying data can be found in S1 Data.
(PNG)

S20 Fig Effect of AHA, CBS, cmpd4 and cmpd8 on the survival of AGS with *H*. *pylori* infection. AHA, CBS, cmpd4, and cmpd8 (10 μM) were administrated to AGS cells with *H*. *pylori* infection. AHA showed little effect on inhibition of activity of *H*. *pylori*; CBS could protect AGS from cytotoxicity of *H*. *pylori*. As expected, low viability of AGS with the supplement of cmpd4 and cmpd8 was observed due to the toxicity of small compounds to mammalian cells. The underlying data can be found in S1 Data.
(PNG)

## Abbreviations

2-DE: 2-dimensional gel electrophoresis
AHA: acetohydroxamic acid
CBS: colloidal bismuth subcitrate
FAK: Focal Adhesion Kinase
FRET: fluorescence resonance energy transfer
GPR55: G protein-coupled receptor 55
GTP: guanosine-5'-triphosphate
HCl: hydrochloric acid
HPC: high-performance computing
HTS: high throughput screening
IC: inhibitory concentration
IL1β: Interleukin 1 beta
LMCT: ligand-to-metal charge transfer
MIC: minimum inhibitory concentration
MOI: multiplicity of infection
NTA: nitrilotriacetic acid
RGS7: Regulator of G-protein Signaling 7
SID: substance identifier number
UFF: universal force field
UV-vis: ultraviolet-visible
