# Supplementary material for: Metallochaperone UreG serves as a new target for design of urease inhibitor: A novel strategy for development of antimicrobials
Source: PLoS Biol. 2018 Jan 10;16(1):e2003887. doi: 10.1371/journal.pbio.2003887 (PMC5779714; doi:10.1371/journal.pbio.2003887)
Supplement: S1 Table — The mutation sites are indicated in red. (DOCX) [file pbio.2003887.s002.docx]

**Table S1** List of primers for plasmid construction. The mutation sites are indicated in red

| **Name** | **Sequence (5’ – 3’)** | **Restriction Endonucleases** |
| --- | --- | --- |
| UreG-For | GGCATATGGTAAAAATTGGAGTTTGTG | *Nde*I |
| UreG-Rev | CGGAATTCCTAATCTTCCAATAAAGCGTTGC | *Eco*RI |
| UreG-C48A-For | GAGTTTATGGCTAAAAATTCGGTG |  |
| UreG-C48A-Rev | TGCGTCTTCTTTCGTGTAAAATAAC |  |
| UreG-C66A-For | CAGGAGGCGCTCCGCACACGGCTATTAG |  |
| UreG-C66A-Rev | TTTCTACGCCAATGATCCTCTCTCGTGG |  |
| UreA2H-For | GGCATATGAAACTCACCCAAAAAGAGTTAG | *Nde*I |
| UreA2H-Rev | GGGAATTCTCAAACCTTTTGCGTGGTGGTTTG | *EcoR*I |
| UreA2HΔG-For | GATCGCTTGGATCAAGCGCAACGCTTTA |  |
| UreA2HΔG-Rev | TCAAGACATATAAAGGCGCGAGTATAAAC |  |
| UreGΔNKXD-For | GCTTGTCATCGCTGCGATTGCTTTAGCC |  |
| UreGΔNKXD-Rev | AAGTCTGAGCGCGTGATTCCTGGCC |  |
|  |  |  |
