# Supplementary figures and images for: Metallochaperone UreG serves as a new target for design of urease inhibitor: A novel strategy for development of antimicrobials
Source: PLoS Biol. 2018 Jan 10;16(1):e2003887. doi: 10.1371/journal.pbio.2003887 (PMC5779714; doi:10.1371/journal.pbio.2003887)

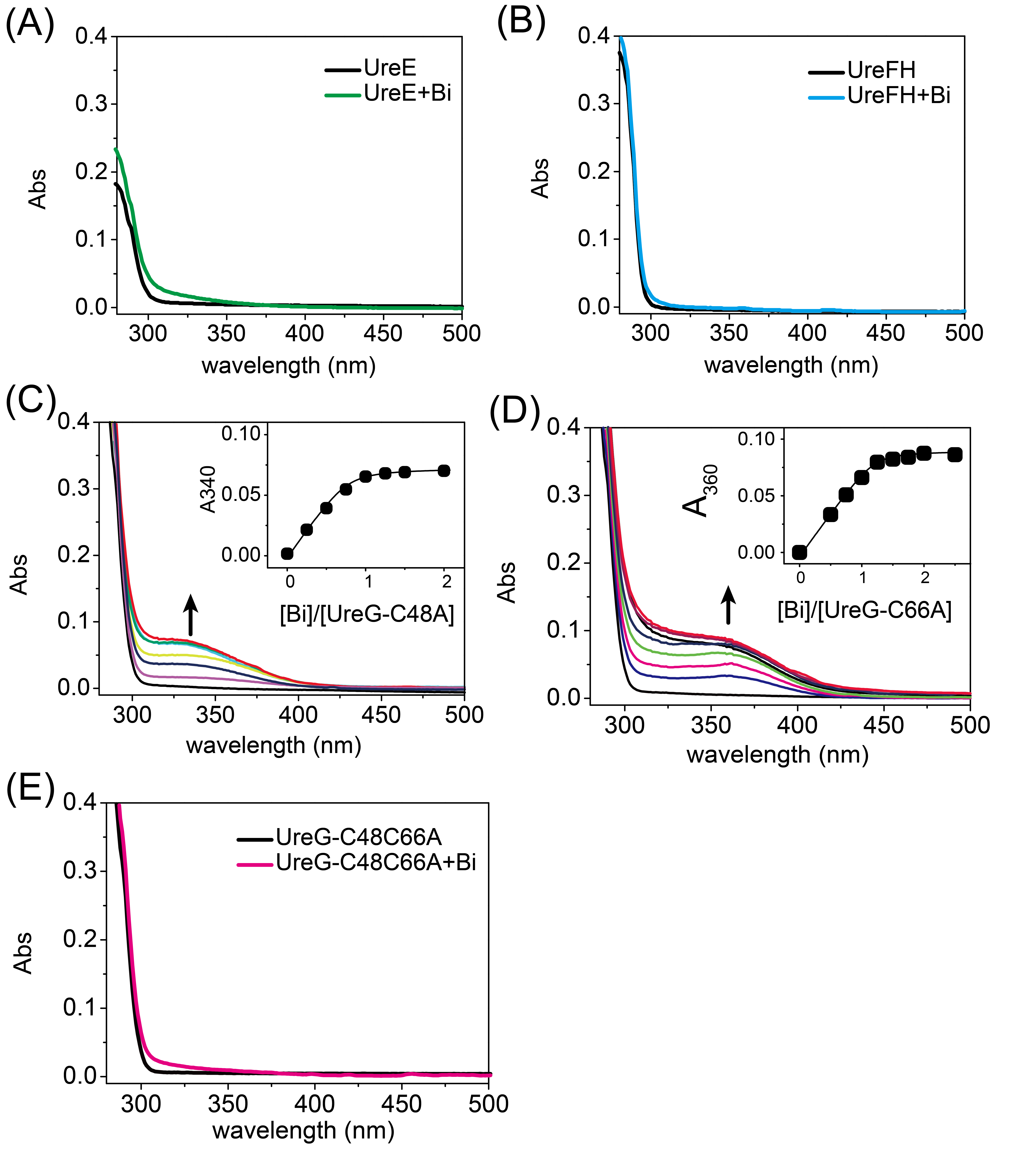

Supplement: S1 Fig — (A) UreE, (B) UreFH complex, (C) UreG-C48A, (D) UreG-C66A, and (E) UreG-C48C66A. The peaks at approximately 340 nm and approximately 360 nm indicated Bi(III) binding to Cys66 and Cys48 (in UreG-C48A and UreG-C66A), respectively (C, D). the shift of absorption peaks may be due to the different coordination or circumstances. (PNG) [file pbio.2003887.s003.png]

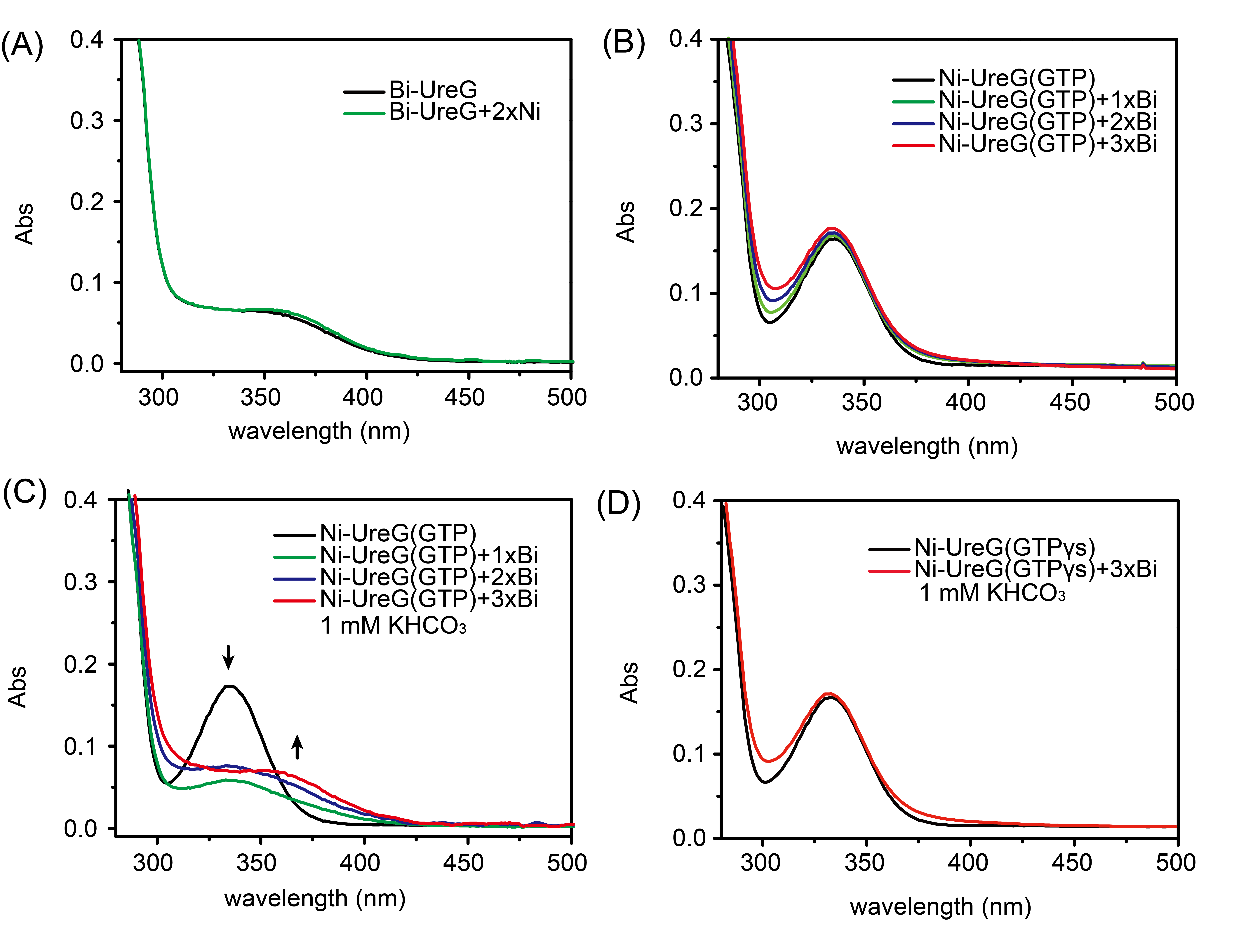

Supplement: S2 Fig — Given the critical role of GTP and Mg(II) in Ni-binding of UreG, UV spectroscopic studies were carried out in HEPES buffer containing 100 μM GTP and 1 mM MgSO4. (A) UV spectra of Bi-UreG upon addition of zero to two molar equivalents of Ni(II) ions. (B) UV spectra of Ni-UreG upon incubation with up to three molar equivalents of Bi(III) ions. It is noted that addition of Bi(III) to Ni-UreG did not suppress the characteristic peak at approximately 337 nm (π(S)(Cys)→Ni(II) LMCT), while the LMCT peak of π(S)(Cys)→Bi(III) (approximately 350 nm) remained undetectable, indicating the lack of Bi(III) coordination to UreG protein when the metal binding site is preloaded with Ni(II). (C) UV spectra of Ni-UreG upon incubation with up to three molar equivalents of Bi(III) ions in the presence of GTPase-activating element KHCO3 (1 mM). Gradual addition of Bi(III) to UreG solution led to a decrease in intensity of the peak at approximately 337 nm and the emergence of a peak at approximately 350 nm, indicative of the simultaneous replacement of Ni(II) ions by Bi(III) on UreG protein. (D) UV spectra of Ni-UreG(GTPγs) upon incubation with up to three molar equivalents of Bi(III) ions in the presence of KHCO3 (1 mM). The characteristic Ni-binding peak was not disturbed, while the typical Bi coordination peak was unnoticeable even after the supplementation of excess Bi(III). It is noted that Bi(III) only disturbs UreG dimer at its GTPase transition state (i.e., in the presence of GTPase-activating elements), but not at its stable Ni, GTP-bound state. (PNG) [file pbio.2003887.s004.png]

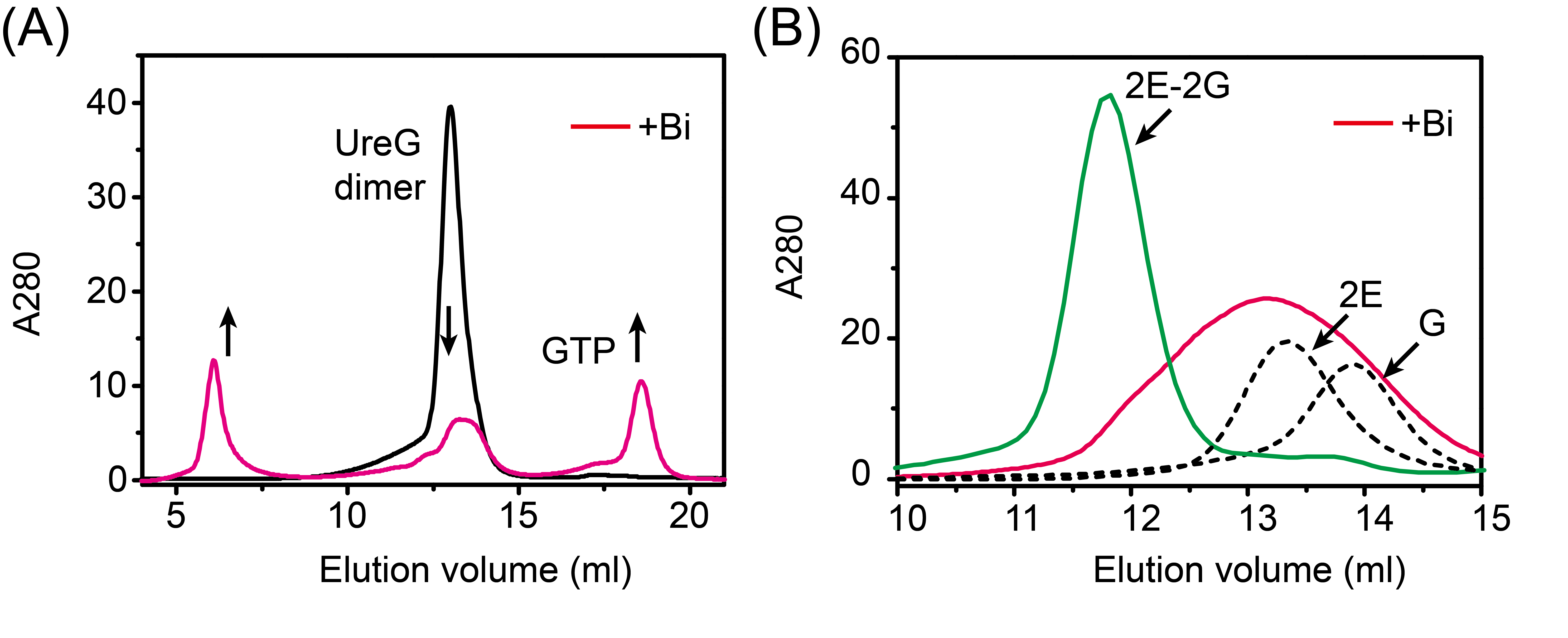

Supplement: S3 Fig — (A) Oligomeric states of Ni-UreG with (red curve) or without (black curve) two molar equivalents of Bi(III) treatment in the presence of KHCO3 (1 mM). (B) Oligomeric states of UreE-UreG complex (2E-2G) with (red curve) or without (green curve) molar equivalents of Bi(III) treatment. (PNG) [file pbio.2003887.s005.png]

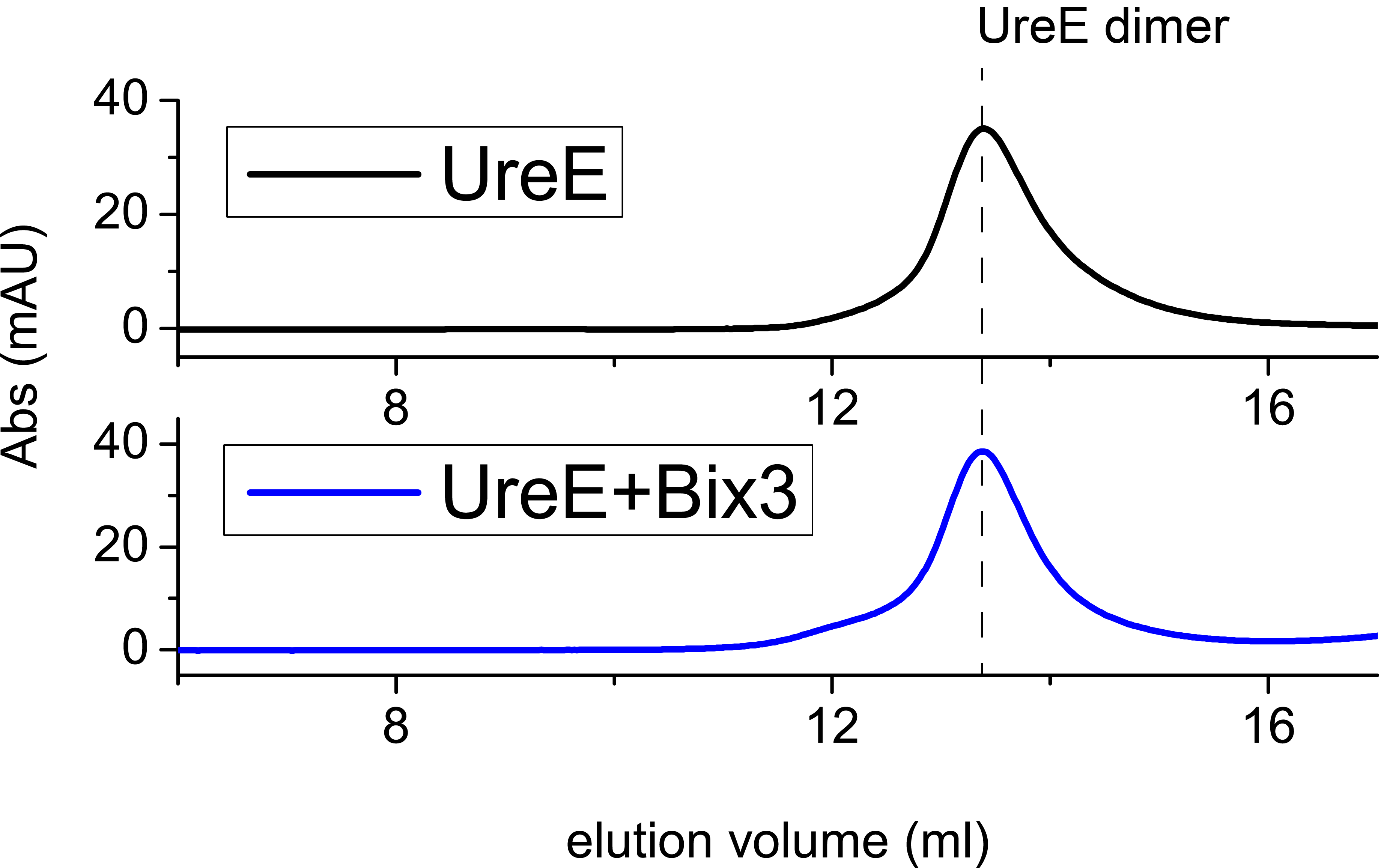

Supplement: S4 Fig — Apo-UreE was eluted at approximately 13.5 ml corresponding to its dimeric form. Incubation with three molar equivalents of Bi(III) has little effect on the UreE dimer. (PNG) [file pbio.2003887.s006.png]

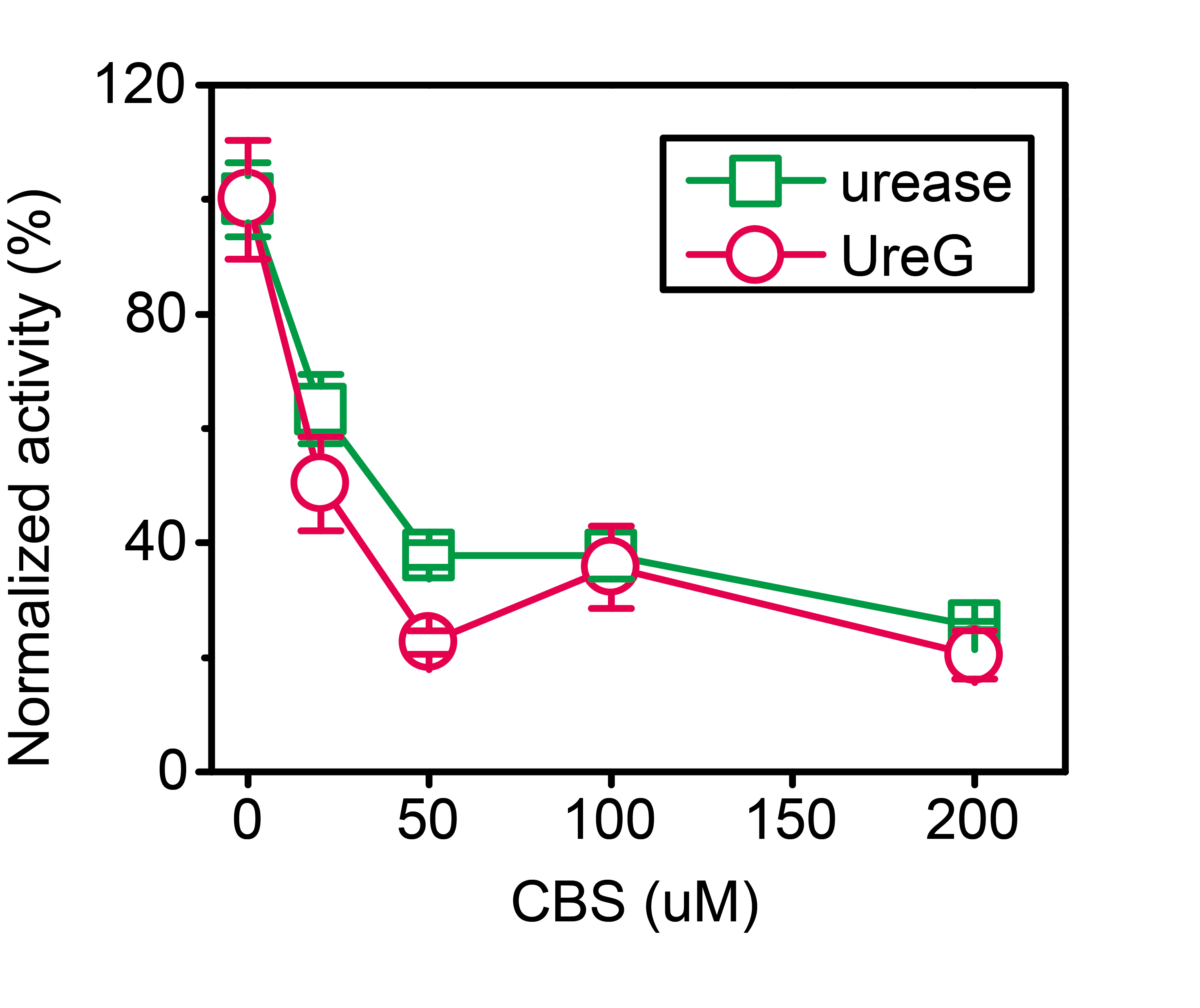

Supplement: S5 Fig — The ureG gene (plasmid pET32a-ureG) was complemented to E. coli cells harboring plasmid pHP8080ΔG; the expression of ureG gene was induced by 100 μM IPTG. After growth, with the addition of gradient amounts of CBS in cultured medium, the GTPase and ureolytic activities of E. coli cell lysate were monitored simultaneously. As UreG was overexpressed, the GTPase activity of E. coli cell lysate was associated with UreG. For convenient comparison, the activities of enzymes (GTPase and urease) in the samples without CBS treatment were set as 100%; the activities of the negative control (without addition of cell lysate into the reactions) were set as 0. The underlying data can be found in S1 Data. (PNG) [file pbio.2003887.s007.png]

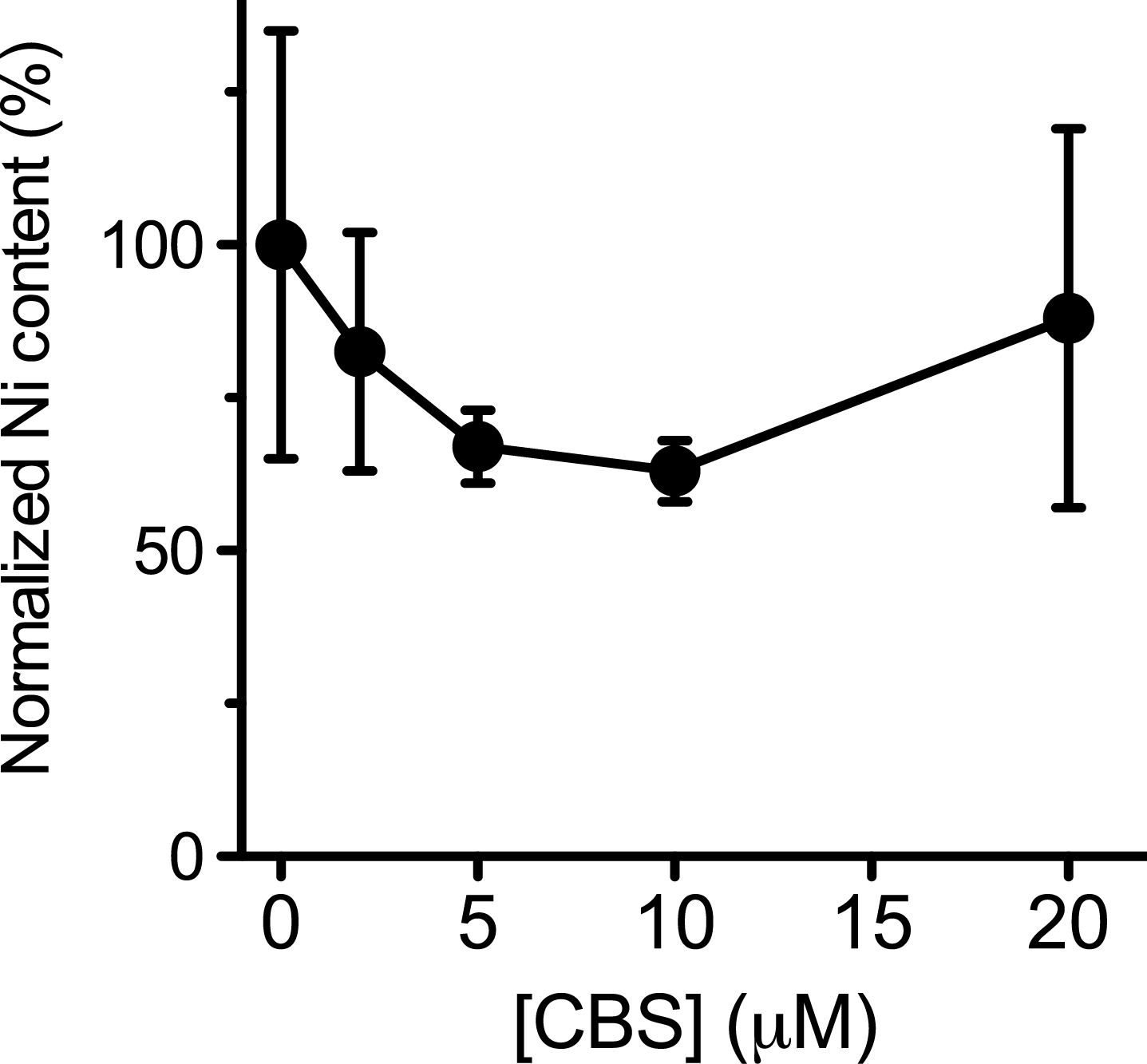

Supplement: S6 Fig — H. pylori was cultured with or without supplementation of Bi(III) to medium. After harvest and washing, the Ni content of H. pylori cells was determined by ICP-MS sequentially. For convenient comparison, the Ni contents in the samples without CBS treatment were set as 100%. As H. pylori has an efficient system for nickel sequestration, H. pylori was cultured without supplementation of excess Ni(II) in cultured medium. The underlying data can be found in S1 Data. (PNG) [file pbio.2003887.s008.png]

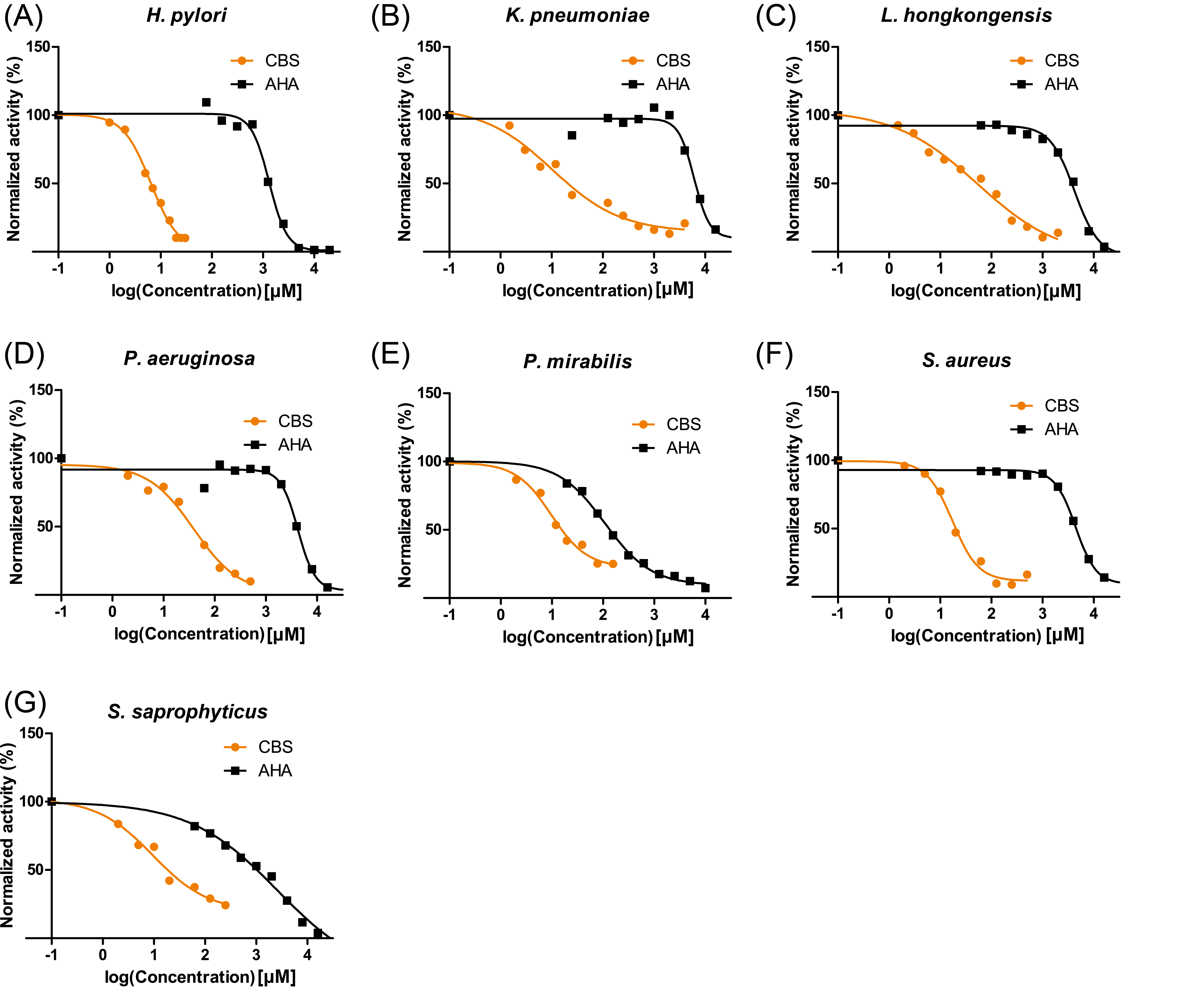

Supplement: S7 Fig — AHA exerts only moderate inhibitory activity against urease with IC50 values at around mM levels, whereas CBS exhibits more potent efficiency on anti-urease activity in bacteria cells. For convenient comparison, the activities of urease in the samples without CBS/AHA treatment was set as 100%; the activities of the negative control (without addition of cell lysate into the reactions) were set as 0. The underlying data can be found in S1 Data. (PNG) [file pbio.2003887.s009.png]

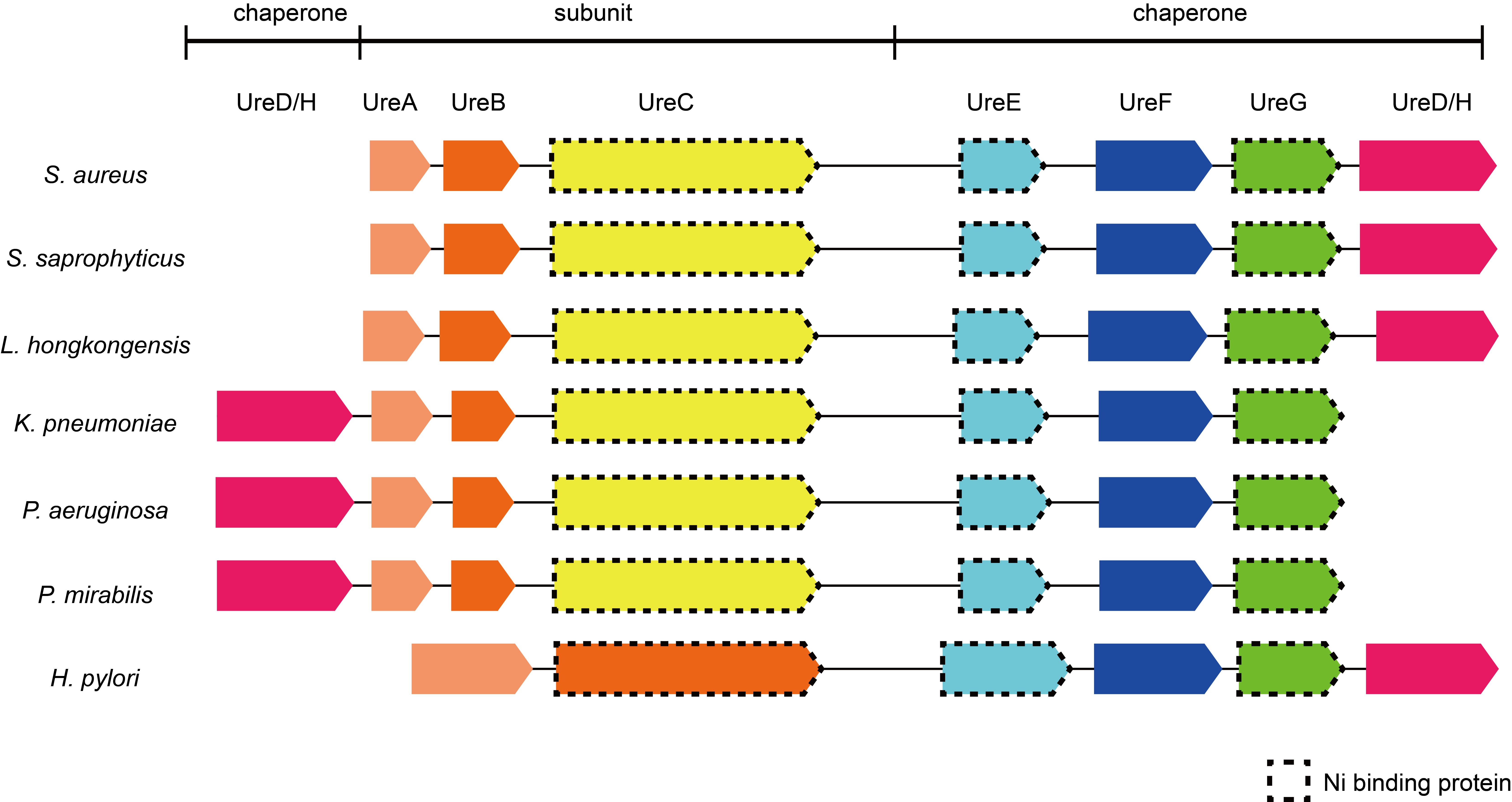

Supplement: S8 Fig — Chaperone UreI, which is not required for urease maturation, has not been illustrated in the figure. (PNG) [file pbio.2003887.s010.png]

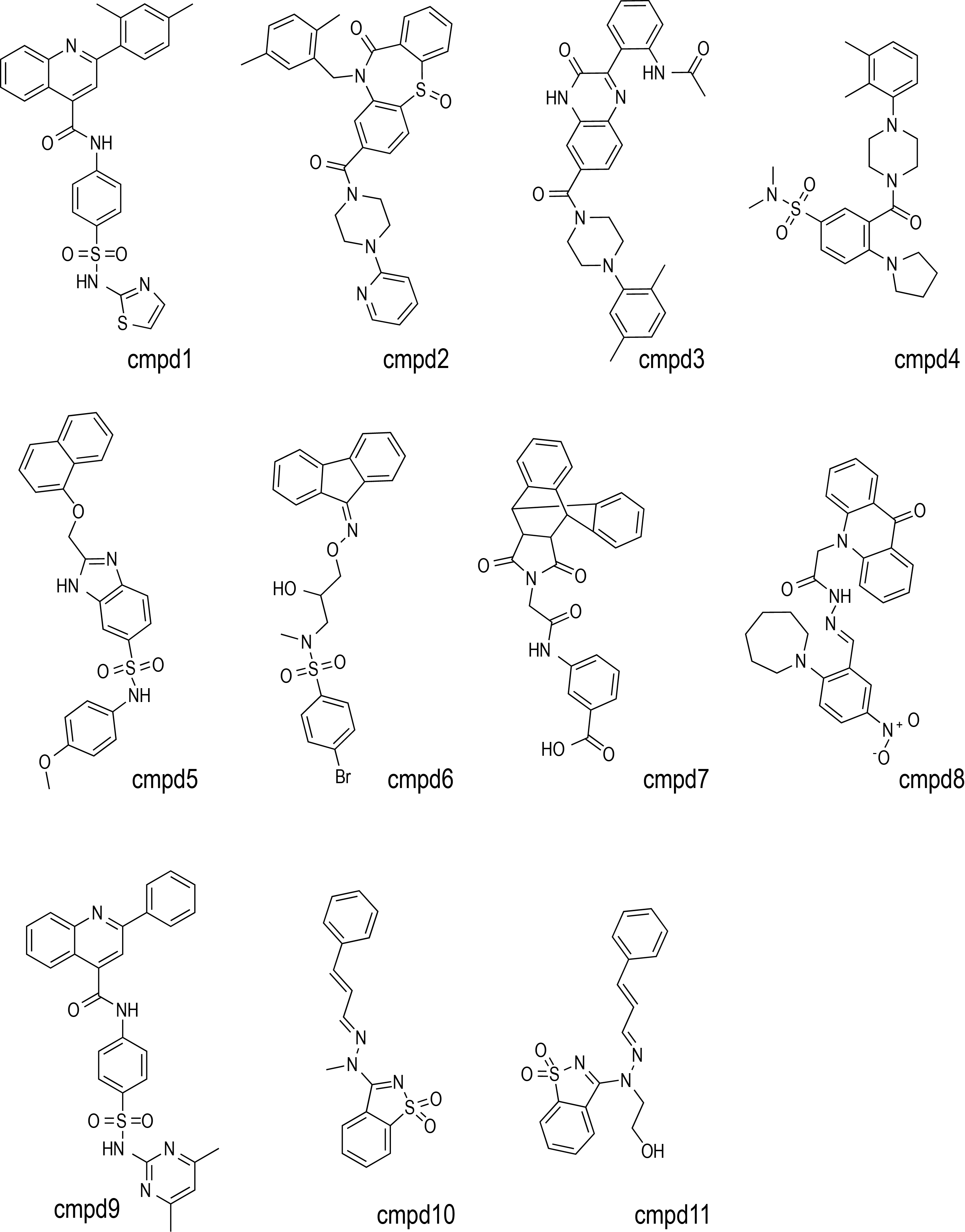

Supplement: S9 Fig — (PNG) [file pbio.2003887.s011.png]

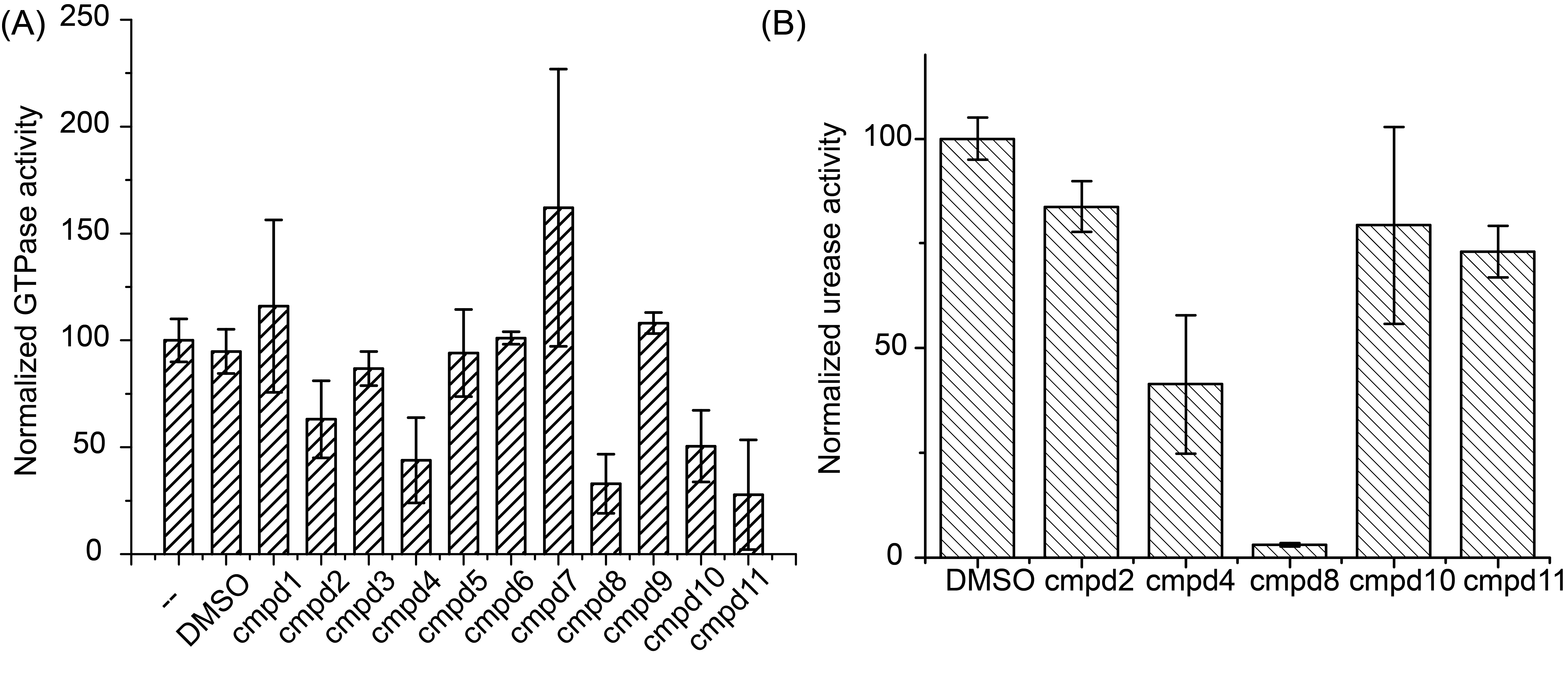

Supplement: S10 Fig — (A) GTPase activity of purified Ni-UreG (5 μM) in the presence of 20 μM small compounds. cmpd7 resulted in serious precipitation in the reaction, which led to the high absorption at 620 nm and false high activity. (B) urease activity of H. pylori cells with the supplement of 20 μM small compounds in cultrued medium. Five tested compounds (cmpd2, cmpd4, cmpd8, cmpd10, and cmpd11) exhibited inhibition effect on the GTPase activity of UreG, whereas cmpd4 and cmpd8 exhibit relatively good anti-urease activity. The underlying data can be found in S1 Data. (PNG) [file pbio.2003887.s012.png]

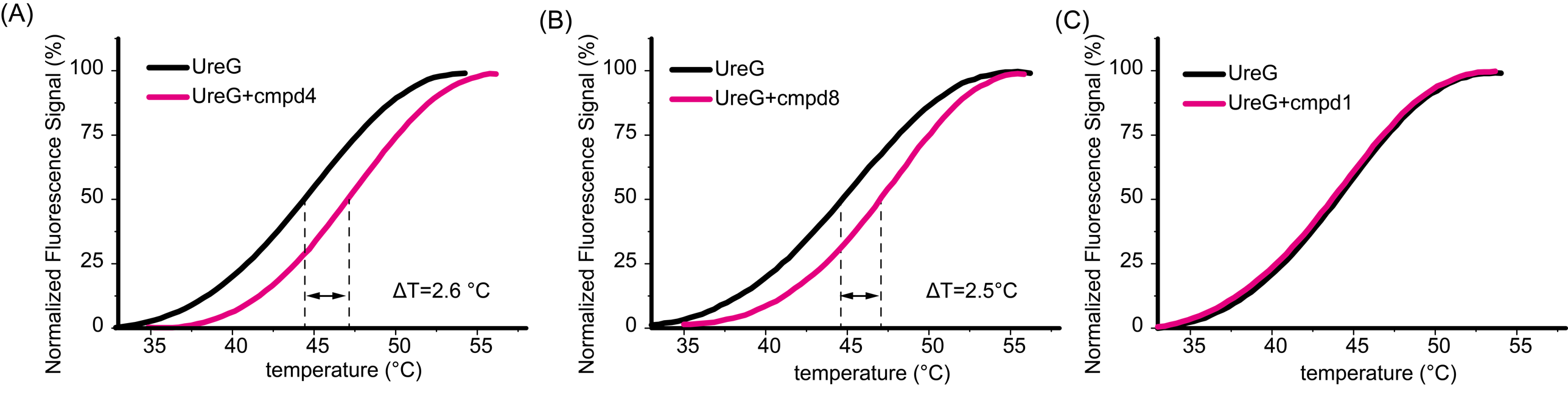

Supplement: S11 Fig — cmpd1, an inactive molecule, was used as a negative control. The underlying data can be found in S1 Data. (PNG) [file pbio.2003887.s013.png]

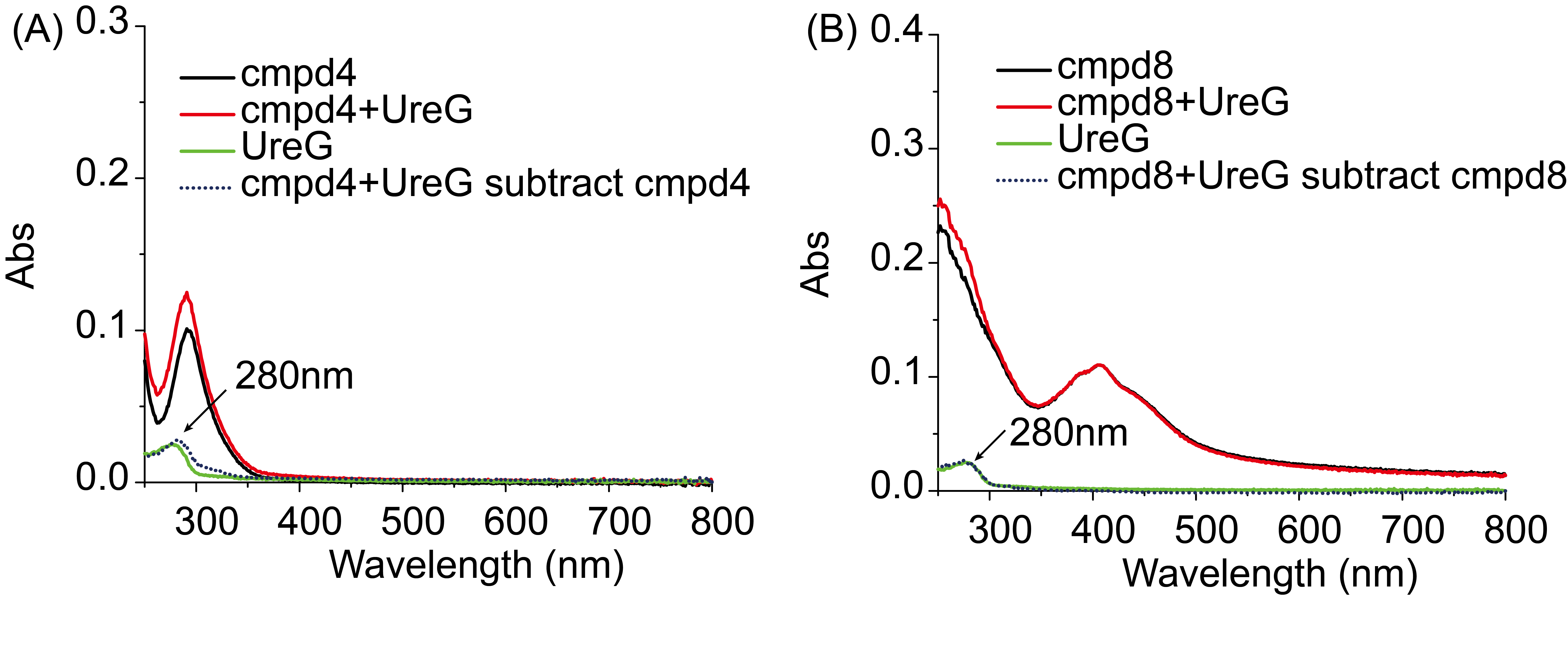

Supplement: S12 Fig — UV-vis spectra of UreG with cmpd4 (A), and cmpd8 (B). Both cmpd4 and cmpd8 (100 μM) alone or in the presence of UreG (1 μM) gave rise to intense absorbance at around 280 nm, whereas the spectra of cmpd4/8-UreG mixture obtained after subtracting the spectra of cmpd4/8 are similar to those of UreG, indicating the presence of cmpd4/8 has negligible effect on the absorption of incident radiation of UreG at 280 nm. (PNG) [file pbio.2003887.s014.png]

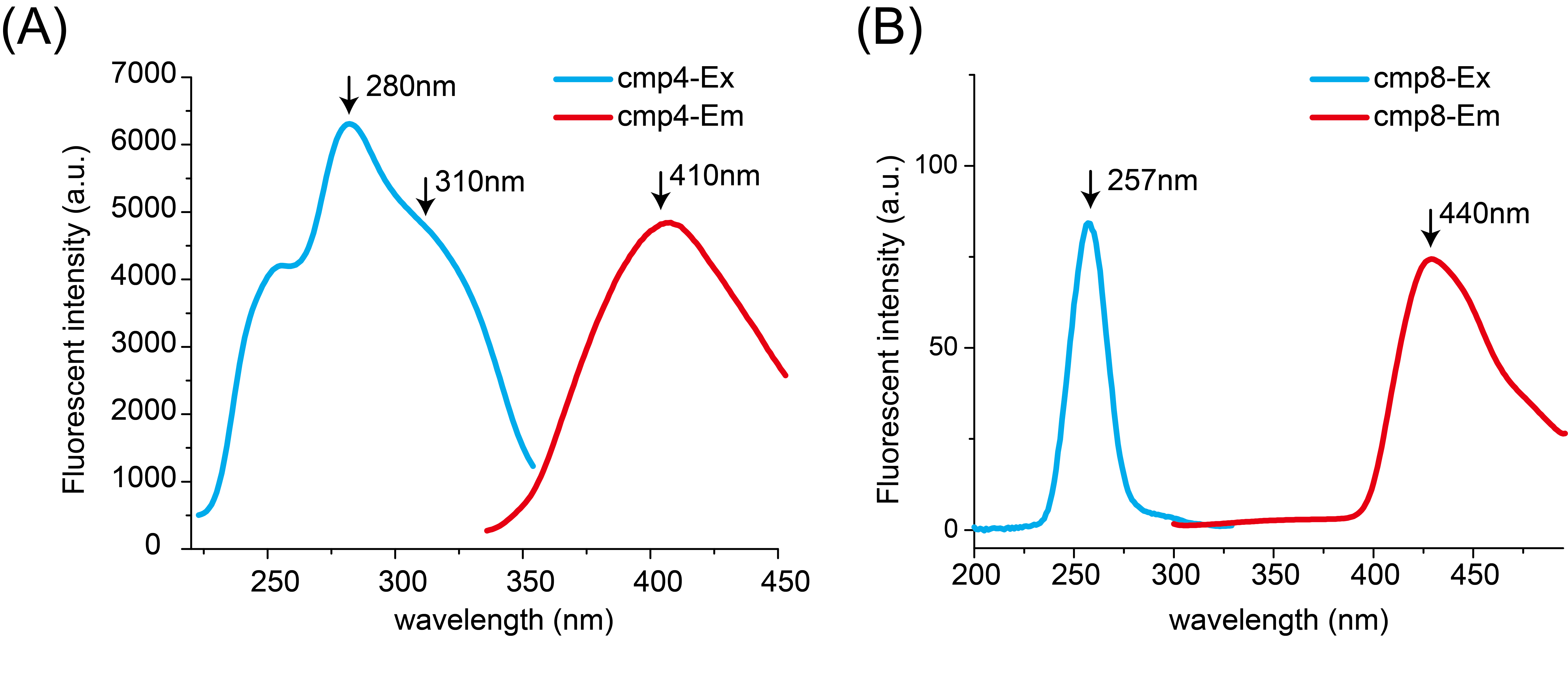

Supplement: S13 Fig — Excitation (blue) and emission (red) spectra of cmpd4 (A) and cmpd8 (B). cmpd4 (100 μM) showed λex = 250, 280, 310 nm and λem = 410 nm, whereas the excitation maxium of cmpd8 (20 μM) was observed at 257 nm and emission maximum at 440 nm. cmpd8 exhibited little excitation at 280 nm; thus, only UreG and cmpd4 have significant fluorescent signals with λex = 280 nm. (PNG) [file pbio.2003887.s015.png]

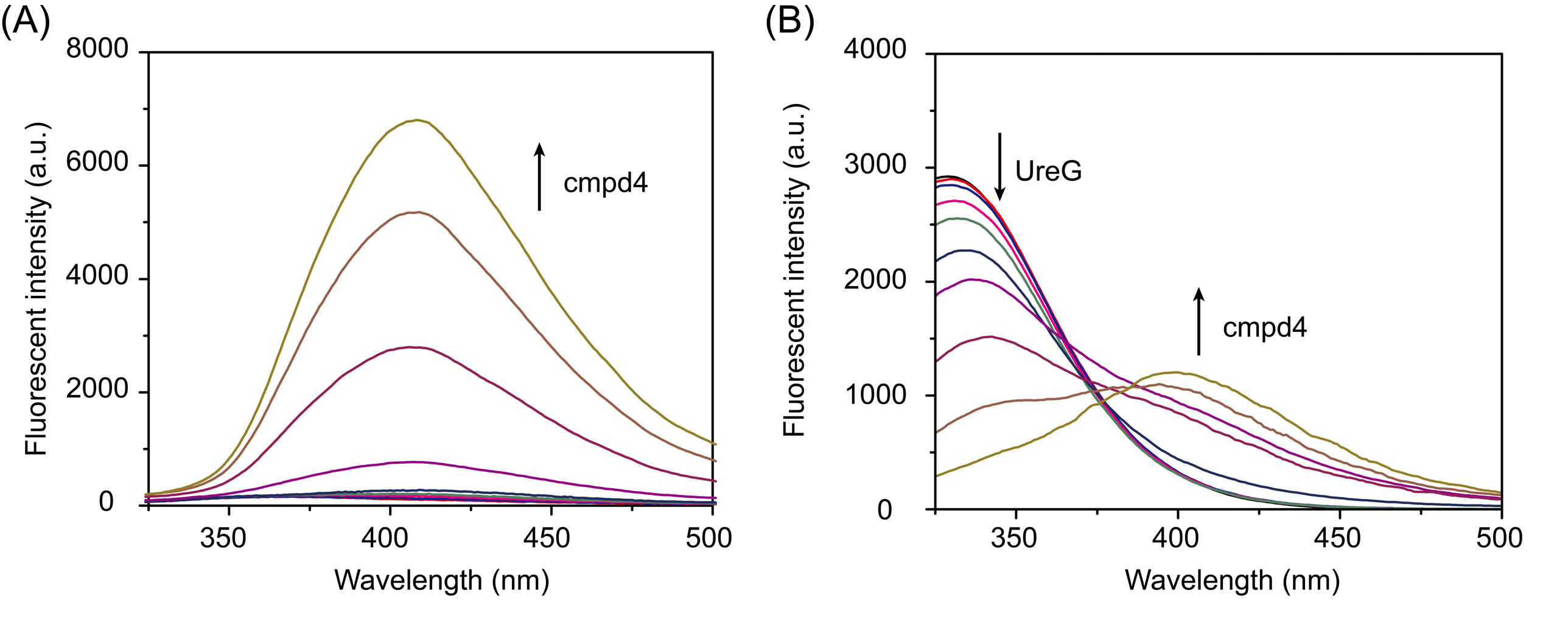

Supplement: S14 Fig — (A) Fluorescence spectra of cmpd4 (B) Titration of cmpd4 produced FRET between UreG and cmpd4. When the mixture of UreG and cmpd4 was excited at 280 nm, the emission intensity at 410 nm (FLUreG+cmpd4, as shown in Fig 4) consisted of two components: the direct emission of cmpd4 and the emission of cmpd4 excited by energy transferred from UreG. Therefore, the FRET emission of cmpd4 (EmFRET) can be determined by EmFRET = FLUreG+cmpd4 − FLcmpd4. (PNG) [file pbio.2003887.s016.png]

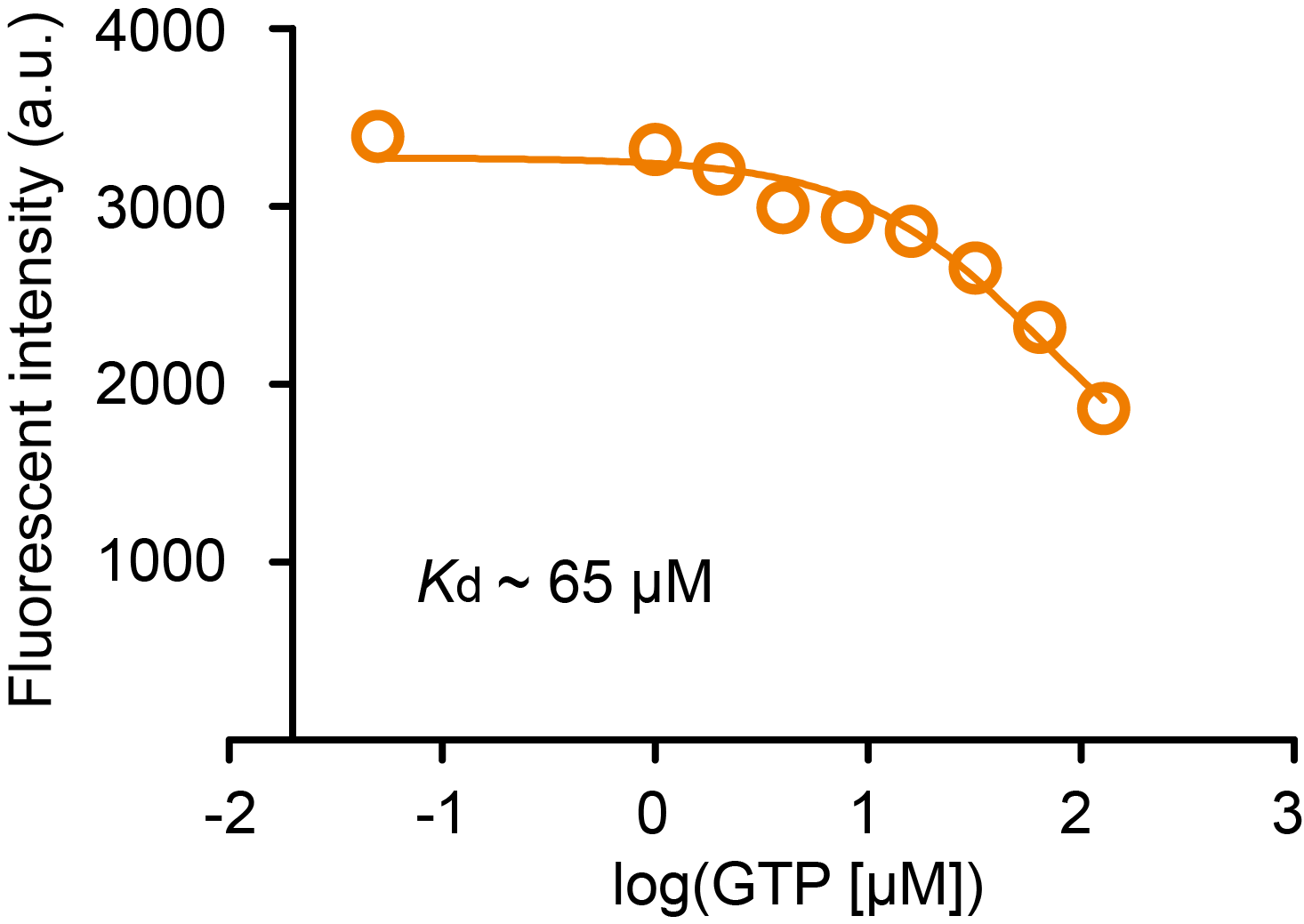

Supplement: S15 Fig — The dissociation constant of GTP to apo-UreG was determined to be ca. 65 μM, consitent with the previous report that apo-UreG does poorly at GTP-binding. The underlying data can be found in S1 Data. (PNG) [file pbio.2003887.s017.png]

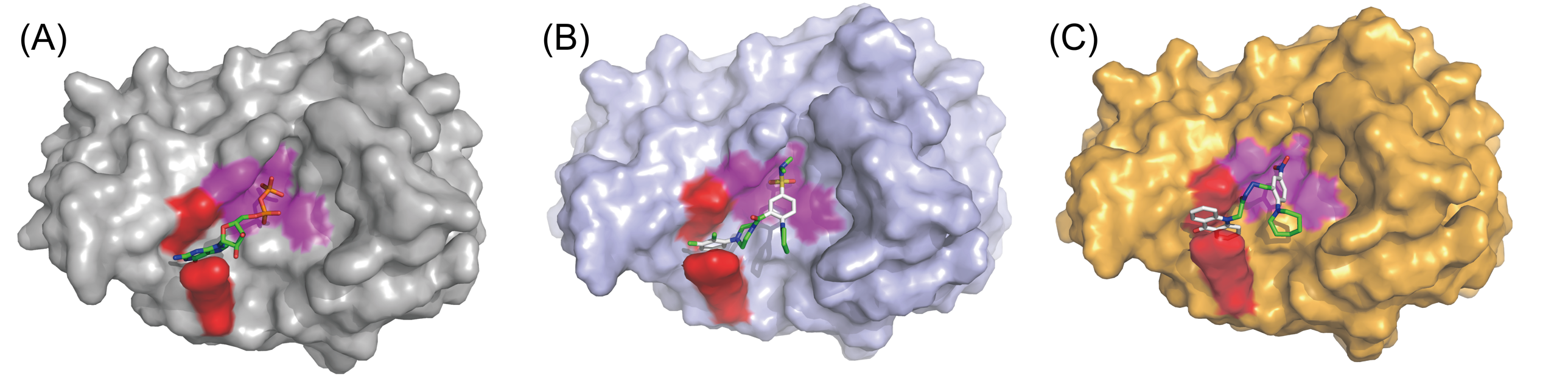

Supplement: S16 Fig — (A) GDP (PDB: 2HI0), (B) cmpd4, and (C) cmpd8. The G1 (P-loop) motif is in magenta, and residues K146 and R179 of UreG are in red, which provide potential hydrophobic interaction with compounds. (PNG) [file pbio.2003887.s018.png]

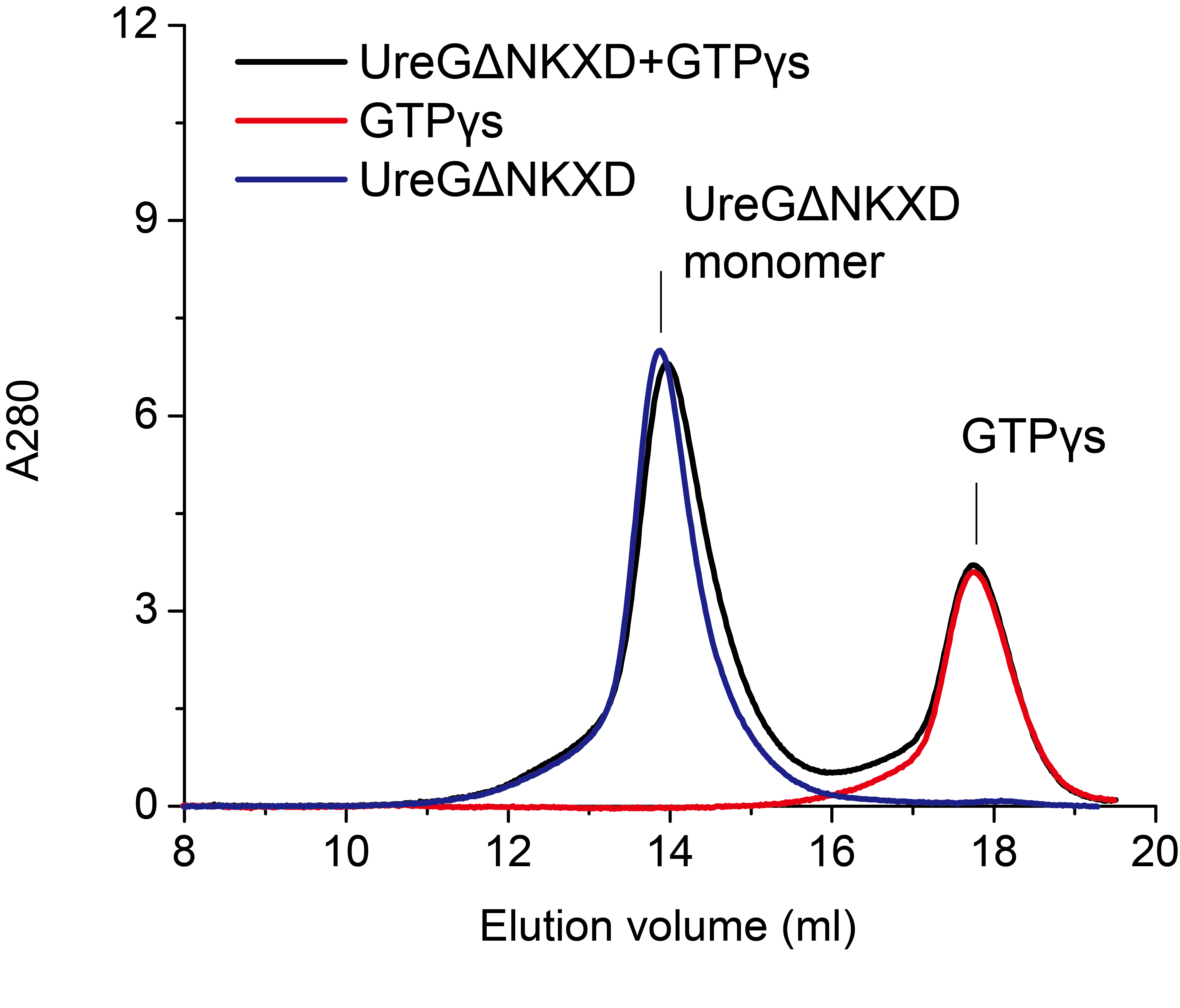

Supplement: S17 Fig — UreG recognizes GTP using the canonical NKXD motif (G4) and is likely to form UreG dimer upon Ni and GTP binding. To examine the effect of the triple mutagenesis (N145A/K146A/D148A) on GTP binding of UreG, UreGΔNKXD (10 μM) was incubated with or without GTPγs (30 μM) in HEPES buffer (20 mM HEPES, 100 mM NaCl, 30 μM NiSO4, 5 mM MgSO4, pH 7.4) and was subjected to gel filtration chromatography. Both UreGΔNKXD samples (with or without GTPγs) were eluted as monomers at approximately 14 ml, and the intensity of peaks at approximately 18 ml corresponding to GTPγs in the presence of UreGΔNKXD remained unchanged compared with that of GTPγs alone, implying that UreGΔNKXD has no GTP binding ability. (PNG) [file pbio.2003887.s019.png]

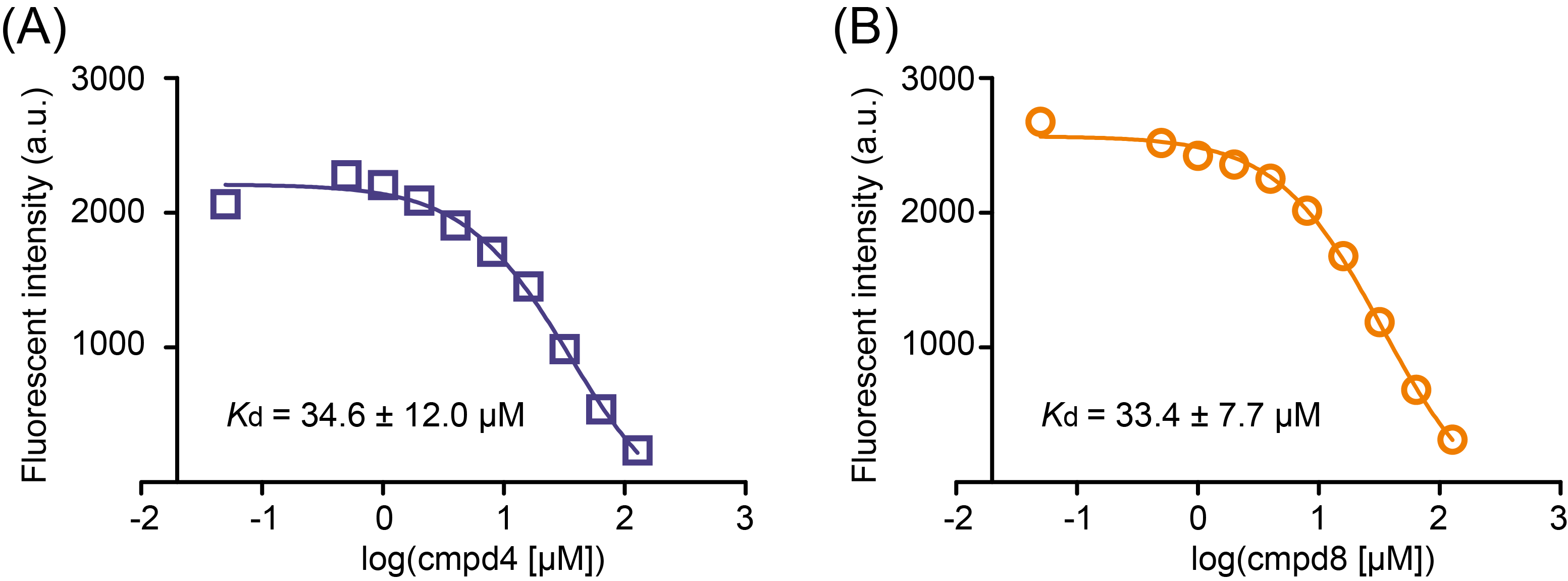

Supplement: S18 Fig — The mutant exhibits lower binding affinity (3 to 4 folds) towards cmpd4/cmpd8 compared to the wild-type UreG. The underlying data can be found in S1 Data. (PNG) [file pbio.2003887.s020.png]

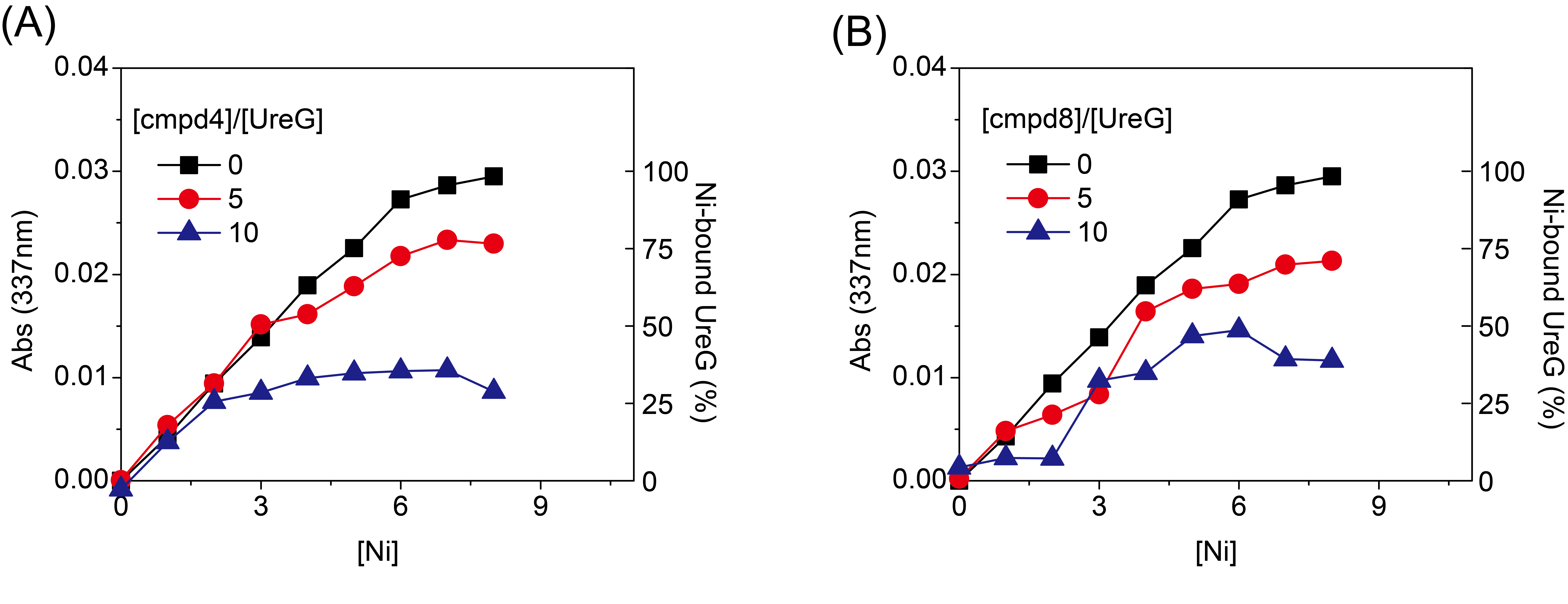

Supplement: S19 Fig — UreG (5 μM) in HEPES buffer (5 μM GTP, 1 mM MgSO4) was titrated with NiSO4 in the presence of cmpd4 or cmpd8 at various concentrations. Titration of nickel ion into UreG samples led to the increasing of absorption at 337 nm, the highest of which was set as 100% for the percentage of Ni-bound UreG. The supplement of cmpd4 and cmpd8 reduced the percentage of Ni-bound UreG. The underlying data can be found in S1 Data. (PNG) [file pbio.2003887.s021.png]

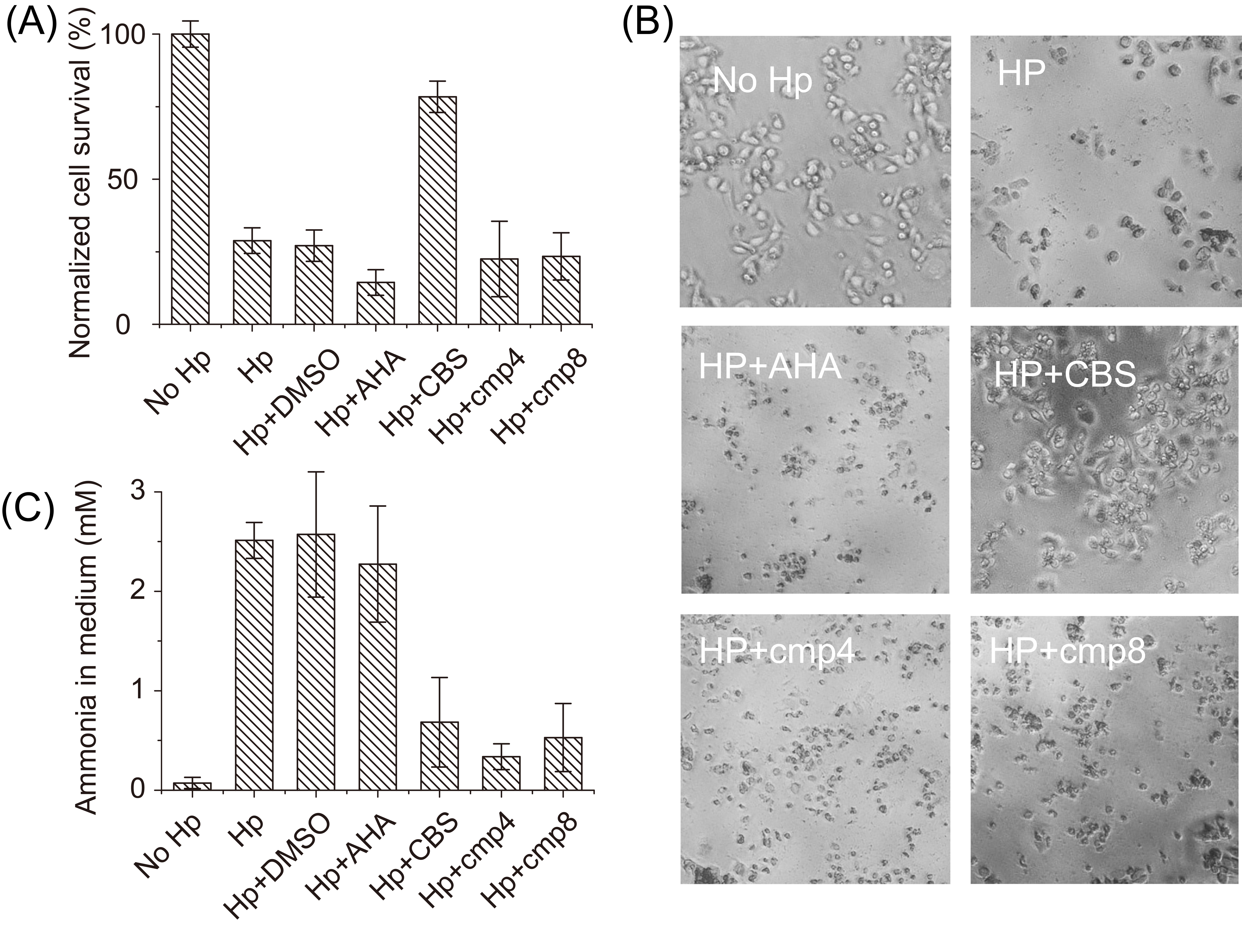

Supplement: S20 Fig — AHA, CBS, cmpd4, and cmpd8 (10 μM) were administrated to AGS cells with H. pylori infection. AHA showed little effect on inhibition of activity of H. pylori; CBS could protect AGS from cytotoxicity of H. pylori. As expected, low viability of AGS with the supplement of cmpd4 and cmpd8 was observed due to the toxicity of small compounds to mammalian cells. The underlying data can be found in S1 Data. (PNG) [file pbio.2003887.s022.png]
